# Differential expression analysis and profiling of hepatic miRNA and isomiRNA in dengue hemorrhagic fever

**DOI:** 10.1038/s41598-020-72892-w

**Published:** 2021-03-10

**Authors:** Layanna Freitas de Oliveira, Amanda Araújo Serrão de Andrade, Carla Pagliari, Leda Viegas de Carvalho, Taiana S. Silveira, Jedson Ferreira Cardoso, André Luiz Teles e Silva, Janaina Mota de Vasconcelos, Caroline Aquino Moreira-Nunes, Rommel Mario Rodríguez Burbano, Márcio Roberto Teixeira Nunes, Eduardo José Melo dos Santos, João Lídio da Silva Gonçalves Vianez Júnior

**Affiliations:** 1 Center for Technological Innovation, Instituto Evandro Chagas, Ananindeua, PA Brazil; 2 Departamento de Patologia, Faculdade de Medicina da Universidade de São Paulo, São Paulo, SP Brazil; 3 Fundação Oncocentro de São Paulo, São Paulo, SP Brazil; 4 Faculdade de Medicina de São José Do Rio Preto, São Paulo, SP Brazil; 5 Instituto de Ciências Biológicas, Universidade Federal do Pará, Belém, PA Brazil; 6 Laboratory of Pharmacogenetics, Drug Research and Development Center (NPDM), Universidade Federal Do Ceará, Fortaleza, CE Brazil

**Keywords:** Pathogens, Dengue virus, miRNAs, Viral infection

## Abstract

Dengue virus causes dengue hemorrhagic fever (DHF) and has been associated to fatal cases worldwide. The liver is one of the most important target tissues in severe cases, due to its intense viral replication and metabolic role. microRNAs role during infection is crucial to understand the regulatory mechanisms of DENV infection and can help in diagnostic and anti-viral therapies development. We sequenced the miRNome of six fatal cases and compared to five controls, to characterize the human microRNAs expression profile in the liver tissue during DHF. Eight microRNAs were differentially expressed, including miR-126-5p, a regulatory molecule of endothelial cells, miR-122-5p, a liver specific homeostasis regulator, and miR-146a-5p, an interferon-regulator. Enrichment analysis with predicted target genes of microRNAs revealed regulatory pathways of apoptosis, involving MAPK, RAS, CDK and FAS. Immune response pathways were related to NF- kB, CC and CX families, IL and TLR. This is the first description of the human microRNA and isomicroRNA profile in liver tissues from DHF cases. The results demonstrated the association of miR-126-5p, miR-122-5p and miR-146a-5p with DHF liver pathogenesis, involving endothelial repair and vascular permeability regulation, control of homeostasis and expression of inflammatory cytokines.

## Introduction

Dengue virus (DENV) and its four serotypes (DENV-1 to 4) are considered the most important arboviruses that affect humans^1^. DENV is transmitted to humans through bites of mosquitoes of the genus Aedes, especially *Ae. Aegipty* and *Ae. Albopictus* species and 3.9 billion people, in 128 countries, are at risk of infection with dengue viruses^2^. One estimate indicates 390 million dengue infections per year, of which 96 million manifest clinically^1^.

Dengue in the Americas has an endemo-epidemic pattern with outbreaks occurring every three to five years and Brazil is among the countries most affected by this arboviral disease, with an estimative of 13.6 million cases between 1990 and 2017^3^.

The clinical manifestations associated to dengue disease ranges from milder forms, denominated as classical dengue fever (DF), to severe forms named as dengue hemorrhagic fever (DHF). DHF is basically characterized by plasma leakage syndrome that can often lead to death^4^. According to the Brazilian Ministry of Health, between epidemiological week (EW) 1 and EW 32 of 2020 (08/08/2020) a total of 918,773 suspected cases were reported in the country (incidence of 4372 cases/10,000 inhabitants), whose 735 were confirmed as dengue severe cases leading to 457 deaths^5^.

Infections caused by DENV can affect several organs, the commonest being the liver. Hepatic tissue is the site of intense replication and contributes significantly to the increase in viral load that occurs primarily in hepatocytes, where Kupffer cells are the most affected cell type during the infection process^6^. Infection and inflammation in liver leads to the development of an environment with multiple cells expressing IL-6 and TGF-beta, which may be related to apoptosis of hepatocytes^7^. Both IL-6 and TGF-beta are cytokines with key roles in inflammation and are probably associated to increased vascular permeability, plasma leaking and consequent worsening of dengue symptoms^8^. In dengue severe cases an increase of pro inflammatory and vasoactive cytokines as IFN-γ, TNF-α, IL-1, IL-6, IL-8 IL-10, CCL2 (MCP-1) and CCL5 is usually observed. Although not conclusive, hypothesis that hepatic inflammation during dengue infections is related to hepatocytes damage protection and tissue repairing to the homeostasis reestablishment is sustained^9^.

During the immune system action in response to infections, there is an increase in the pathway communication among cells, as well as an intense cytoplasmic microenvironment modification in order to regulate gene expression in response to the agent. The microRNAs (miRNA) are RNA molecules that act in this scenario of RNA interference (RNAi), being key mediators of host response to infectious diseases. The miRNA sequences are single-stranded RNA, 22 nucleotides in length (average), associate to the RNA-induced silencing complex (RISC) and regulate gene expression by binding to the region between the base 2 and 8 (seed region). Its target is the miRNA recognition element (MRE) located at the 3 'UTR of mRNA^10^.

Alternative or imprecise biological processing of miRNA and post-transcriptional modifications may generate sequence variations, the isomiRNAs. The same miRNA locus can give rise to multiple distinct isomiRs, differing in length or sequence composition that might present different functionalities^11^.

Thus, the aim of this study was to characterize the differential miRNA expression profile of human liver tissue from fatal cases of DHF, identify the isomiR profile, and propose the regulatory mechanisms of these molecules in metabolic pathways related to the pathogenesis of the disease.

## Results

### Sequencing

Illumina sequencing of the eleven libraries yielded a total of 57,648,798 reads. Each library generated an average of 5,240,800 reads. After removal of adapters and unidentified nucleotides (N), the mean number of reads per sample was determined at 2,673,069 reads; in all samples, the highest read size frequency was 22 nucleotides, consistent with the documented size to most mature microRNAs (Table 1). One of the DHF samples (Dhf_299) was removed from subsequent analysis since only 10% of the reads passed quality control. The same sample also appeared as an outlier in the MDS plot (Supplementary Figure S1).

**Table 1:** Clinical data and sequencing results per sample. MI: acute myocardial infarction; Dhf: dengue hemorrhagic fever.

Sample ID	Clinical				Sequencing data			
	Age	Sex	Cause of death		Raw reads	Processed reads	Processed reads (%)	Quality
Control-63	44	M	MI, atherosclerosis		1,565,618	724,999	46.3	37
Control-81	48	M	Acute pulmonary edema, MI, systemic atherosclerosis		4,212,073	2,793,822	66.3	37
Control-94	56	M	MI, atherosclerosis		6,192,749	5,263,821	85.0	37
Control-13	58	M	No information		1,498,026	938,826	62.7	37
Control-14	73	F	No information		5,280,226	3,885,399	73.6	37
Dhf-173	52	M	Pleural effusion, trans-infectious hepatitis		7,644,988	3,787,025	49.5	37
Dhf-198	52	F	Pleural effusion and peritoneal, trans-infectious hepatitis, hematemesis		3,001,348	864,836	28.8	37
Dhf-236	41	F	Pleural effusion, ascites		6,079,568	2,349,387	38.6	37
Dhf-255	62	M	Splenomegaly		7,225,149	3,923,213	54.3	37
Dhf-299	43	F	Acute hepatitis, brain edema		6,633,706	717,805	10.8	37
Dhf-347	47	F	Spleen congestion, focus of hemorrhage, hepatic steatosis		8,315,347	4,154,626	50.0	37

### miRNA differential expression analysis: DHF and control samples

We identified a group of 38 differentially expressed miRNAs considering a p-value < 0.05. Considering FDR < 0.05 and log2FC threshold |2|, eight miRNA remained significant: miR-133a-3p, miR-122-5p, miR-10b-5p, miR-204-5p, miR-126-5p, miR-148a-5p, miR-146a-5p and miR-423-5p (Supplementary Table S1, Table 2). Among them, two were up-regulated in DHF liver tissue: miR-133a-3p (log2FC = 6.77; FDR < 0.001) and miR-126-5p (log2FC = 3.09; FDR = 0.007, Supplementary Table S1).

**Table 2:** Top 30 microRNAs most expressed in hepatic tissue of dengue hemorrhagic fever (DHF) and controls, expression is presented by counts per million (CPM).

Ranking	miRNA	Control (average CPM)	miRNA	DHF (average CPM)
1	hsa-miR-122-5p*	225,797.54	hsa-miR-143-3p	93,950.57
2	hsa-miR-192-5p	142,361.14	hsa-miR-192-5p	60,231.03
3	hsa-miR-10b-5p*	134,833.23	hsa-miR-27b-3p	42,784.48
4	hsa-miR-22-3p	99,530.51	hsa-miR-22-3p	42,661.72
5	hsa-miR-143-3p	96,662.53	hsa-miR-21-5p	37,702.97
6	hsa-miR-10a-5p	87,218.63	hsa-miR-10a-5p	26,870.72
7	hsa-miR-30a-5p	65,286.96	hsa-miR-30a-5p	23,868.67
8	hsa-miR-26a-5p	46,657.54	hsa-miR-126-5p*	22,629.93
9	hsa-miR-27b-3p	39,069.49	hsa-miR-26a-5p	22,190.67
10	hsa-miR-148a-3p	32,382.40	hsa-miR-451a	18,251.66
11	hsa-miR-486-5p	29,730.49	hsa-miR-486-5p	16,815.22
12	hsa-miR-146b-5p	26,265.61	hsa-let-7f.-5p	12,382.60
13	hsa-miR-21-5p	17,553.78	hsa-miR-181a-5p	11,090.36
14	hsa-miR-30e-5p	16,693.45	hsa-let-7i-5p	10,764.92
15	hsa-miR-451a	13,438.83	hsa-miR-148a-3p	10,506.86
16	hsa-let-7f.-5p	13,017.50	hsa-miR-30e-5p	9277.09
17	hsa-miR-30d-5p	12,961.10	hsa-miR-16-5p	8057.48
18	hsa-miR-378a-3p	9132.45	hsa-miR-146b-5p	7199.33
19	hsa-miR-92a-3p	8745.08	hsa-miR-101-3p	6530.05
20	hsa-miR-146a-5p*	8548.37	hsa-miR-133a-3p*	6351.99
21	hsa-let-7i-5p	8317.40	hsa-miR-30d-5p	6051.11
22	hsa-let-7a-5p	7247.17	hsa-miR-186-5p	5738.95
23	hsa-miR-92a-3p	7076.30	hsa-miR-26b-5p	5689.11
24	hsa-miR-26b-5p	6166.08	hsa-miR-92a-3p	5455.07
25	hsa-miR-181a-5p	5357.57	hsa-let-7a-5p	5146.89
26	hsa-let-7 g-5p	4898.23	hsa-miR-92a-3p	4914.85
27	hsa-miR-186-5p	4652.75	hsa-miR-126-3p	4757.55
28	hsa-miR-16-5p	4275.93	hsa-let-7 g-5p	4154.16
29	hsa-miR-100-5p	3979.56	hsa-miR-142-5p	3984.70
30	hsa-miR-99a-5p	3712.07	hsa-miR-141-3p	3859.45

*Statistically significant.

Most of the differentially expressed miRNAs were under-expressed in DHF libraries in comparison to the control condition. miR-122-5p showed the highest expression in all controls (log2FC = −6.59; FDR < 0.001), with average CPM = 225,797.2 in controls and 2,336.4 in DHF, that is, it is almost 100 times less expressed in DHF group. miR-10b-5p is the second with the highest expression level in the control group, decreased almost 100 times in DHF (log2FC = −6.167 (FDR < 0.001), followed by miR-146a-5p (log2FC = −2.6476 (FDR = 0.0387), both had high CPM values. The expression of miR-204-5p (log2FC = -5.632; FDR < 0.001), miR-148a-5p (log2FC = −3.135; FDR = 0.007), and miR-423-5p (log2FC = −2.78 and FDR = 0.046) had lower CPM value, nevertheless showed statistical significance (Supplementary Table S1).

**Figure 1:**
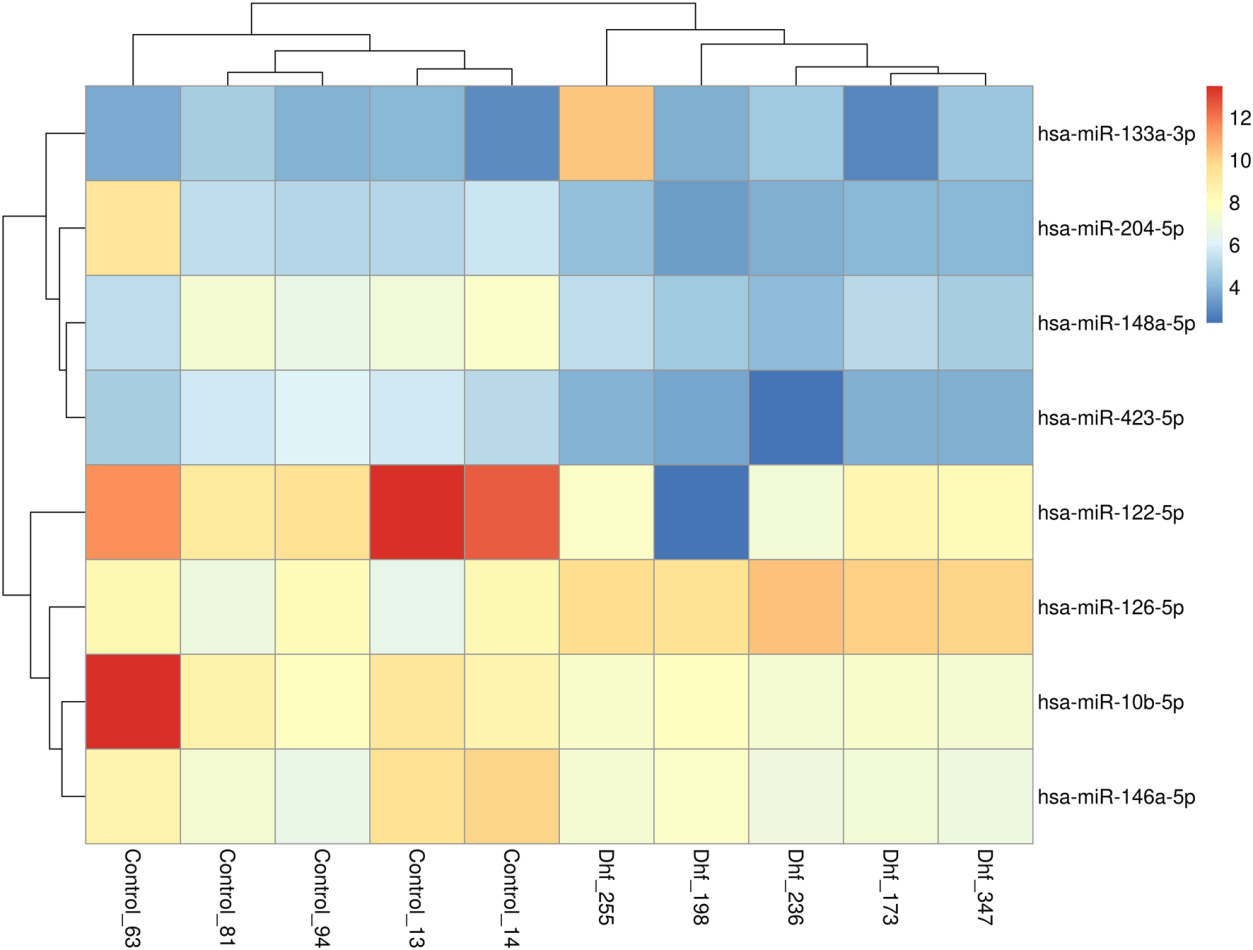
Heatmap analysis of all differentially expressed microRNAs detected in this study using miRNA-seq shows a clear separation of DHF and control samples. The scale bar is in log2(CPM).

The expression of hepatic miRNA could correctly cluster the sample groups in DHF and controls, as showed by the MDS plot, with exception of the sample Dhf_299 which was excluded from the analysis (Supplementary Figure S1). Heatmap analysis based on log2(CPM) of also resulted in distinct clades of DHF and control groups (Fig. 1).

### Target genes prediction and functional analysis

**Figure 2:**
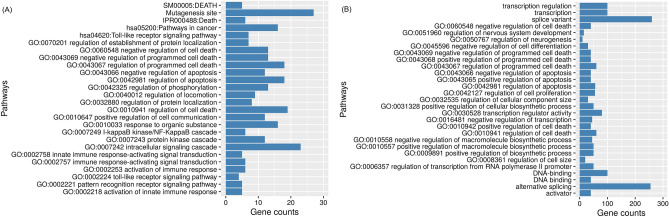
Enrichment analysis of upregulated (**a**) and downregulated (**b**) miRNAs target genes in DHF liver tissue using KEGG, Interpro and GO Terms obtained in DAVID Annotation Tool. All pathways have p-value adjusted by Benjamini method < 0.05.

The target prediction step generated a list of 224 target genes of overexpressed miRNAs in liver were the input dataset to functional annotation. The enrichment analysis identified 26 pathways with significant p-value < 0.05 after Benjamini adjustment: nine pathways of apoptosis and cell cycle regulation; seven related to the immune response including pathways activated by NF-κβ, the other ten pathways are mainly related to cell signaling and signal transduction (Fig. 2A, Supplementary Table S2). Functional analysis involving target genes of underexpressed miRNAs in DHF group was carried out with 570 target genes, from six downregulated miRNAs, obtained in the three databases and resulted in 208 pathways, with the predominance of cell death pathways (Fig. 2B, Supplementary Table S3).

### isomiRNA differential expression analysis

**Figure 3:**
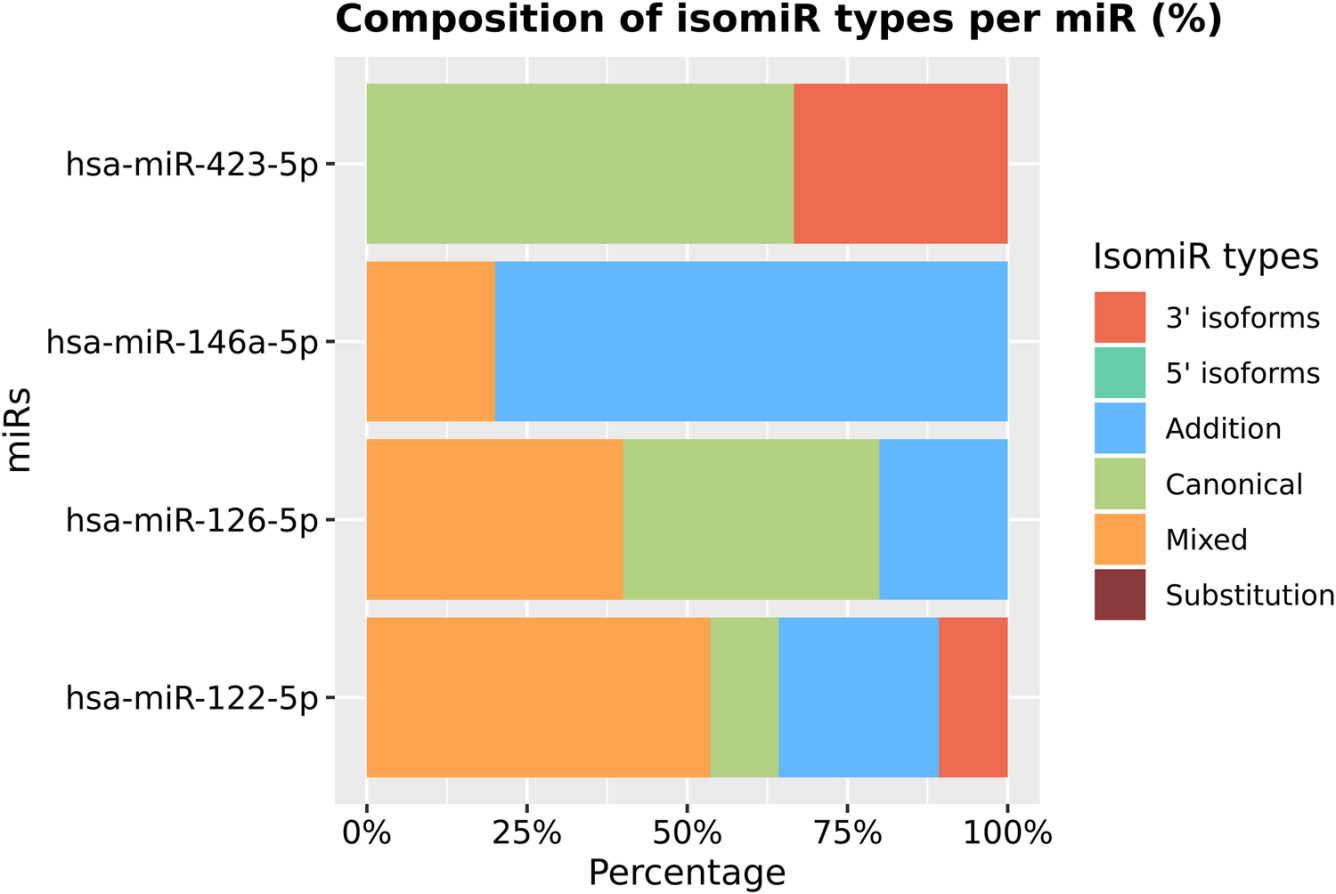
isomiRs fraction for the miRNAs miR-122-5p, miR- 126-5p, miR-146a-5p and miR-423-5p.

At total, we detected 5500 differentially expressed isomiRs, however only 188 passed our Log2FC and p value criteria. Of these, 66 were canonical sequences and 122 were isomiRs. From the 8 miRNAs previously associated with DHF in hepatic profile: four presented isomiRs (hsa-miR-122-5p, hsa-miR-126-5p, hsa-miR-146a-5p and hsa-miR-423-5p), two (hsa-miR-204-5p and hsa-miR-148a-5p) presented only their canonical sequence and two (hsa-miR-133a-5p and hsa-miR-10b-5p) were not considered differentially expressed in our samples. The isomiRs fractions of the each four miRNAs associated with DHF, described here, is demonstrated in Fig. 3.

### Top 30 differentially expressed isomiRs

The 30 most significant isomiRs in the DE analysis are described in Table 3 as well as their respective categories and relevant statistical data. The counts were normalized in CPM and represent the group mean and the top was arranged from lowest to highest adjusted p values. Concerning the software isomiRs classification, sequences containing nt addition (uppercase) or deletion (lower case) in the UTR ends are classified as 5′ (**t5**) or 3′ isoforms (**t3**). Addition anywhere else is **ad**, substitution is **mm** and if there are a discrepancy in the 5′ end 2-8nt is **seed** represented by the nt position and the modification. The canonical sequence is **ref**. For example, the mature sequence of miR-122-5p is if the sequence of the anotated miRNA is UGGAGUGUGACAAUGGUGUUUG. The annotation miR-122-5p.iso.t3:T corresponds to an addition (T) in the 3′end, resulting in the sequence UGGAGUGUGACAAUGGUGUUUG**T**.

**Table 3:** Top 30 differentially expressed isomiRs in the hepatic tissue.

Top IsomiRs		Log2FC	Adjusted p-value
1	miR-122-5p.iso.t3:T	− 8.96	4.00E−09
2	miR-122-5p.iso.ad:A	− 7.60	1.24E−05
3	miR-122-5p.iso.ad:C	− 6.79	1.47E−04
4	miR-122-5p.iso.ad:G	− 6.09	1.11E−04
5	miR-122-5p.iso.seed:0.ad:AT	− 7.70	8.43E−07
6	miR-122-5p.iso.t3:g.ad:A	6.34	1.24E−05
7	miR-122-5p.iso.t3:g.ad:AT	− 5.71	3.73E−06
8	miR-122-5p.iso.t3:g.ad:T	3.90	2.27E−05
9	miR-122-5p.iso.t3:g.ad:TT	− 7.33	3.30E−05
10	miR-122-5p.iso.t3:T.ad:T	− 5.22	6.43E−06
11	miR-122-5p.iso.t3:tg.ad:GA	− 6.37	4.13E−05
12	miR-122-5p.iso.t3:tg.ad:GT	− 8.19	3.32E−07
13	miR-122-5p.iso.t5:t.t3:T	− 7.84	3.79E−06
14	miR-126-3p.ref	3.21	9.92E−05
15	miR-126-5p.ref	− 8.00	3.73E−06
16	miR-126-5p.ref	4.60	3.76E−05
17	miR-142-3p.iso.t3:ga.mm:12TC	5.51	3.18E−04
18	miR-144-3p.iso.t3:ct.ad:TT	6.05	8.43E−07
19	miR-148a-5p.ref	− 3.41	3.66E−05
20	miR-16-5p.iso.t3:cg.ad:TG	4.83	6.98E−04
21	miR-181b-5p.ref	4.41	1.47E−04
22	miR-21-5p.iso.ad:T	3.98	4.13E−05
23	miR-23a-3p.iso.mm:10TC	6.49	4.13E−05
24	miR-23b-3p.iso.mm:10TC	6.63	3.00E−05
25	miR-26b-5p.iso.t3:t.ad:C	3.64	6.24E−04
26	miR-27a-3p.ref	3.43	6.19E−04
27	miR-320a.iso.t3:a.ad:T	− 5.30	6.98E−04
28	miR-423-5p.ref	− 6.04	8.43E−06
29	miR-7704.iso.ad:GGC.mm:19CG	− 6.82	1.85E−04
30	miR-99a-5p.iso.seed:5TC.t3:tg	− 6.51	1.70E−05

### DE divergent isomiRs

**Figure 4:**
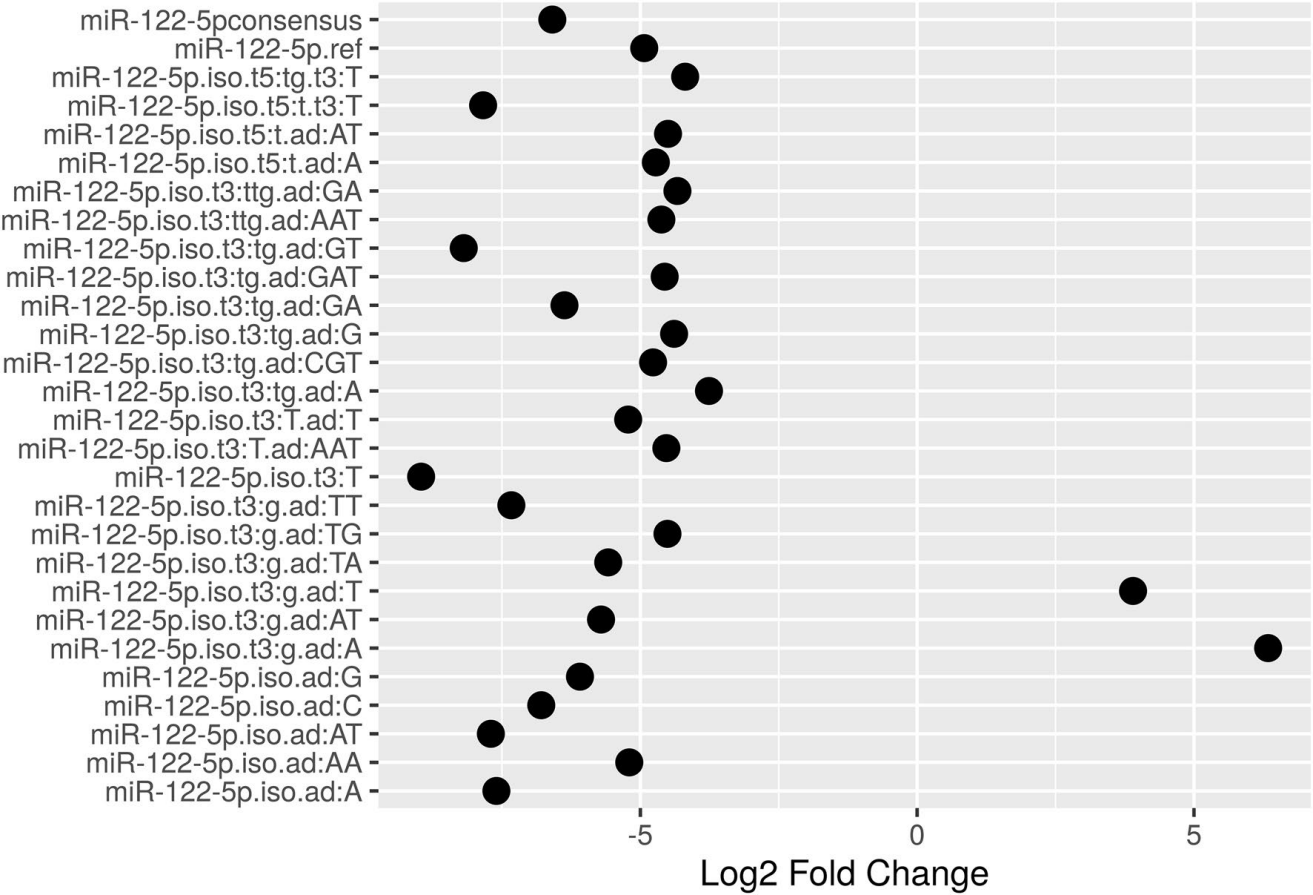
Log2FC for miRNA 122-5p isoforms. The first line, miR-122-5p consensus, demonstrate the Log2FC mean for all miR-122-5p isoforms found in the samples.

Two miRNAs presented divergent expression among their isoforms. The human miRNA 122-5p obtained 28 isoforms differentially expressed, and the miRNA 99a-5p eight found isoforms. The expression pattern and the divergences for the miRNA 122-5p are exposed in Fig. 4.

## Discussion

Our miRNA differential expression analysis in DHF and Control liver samples showed eight significant differentially expressed miRNA (miR-133a-3p, miR-122-5p, miR-10b-5p, miR-204-5p, miR-126-5p, miR-148a-5p, miR-146a-5p and miR-423-5p). In addition, our isomiR analysis showed 65% of all mature miRNA sequence as isomiRs, including isomiRs for miR-122-5p, miR-126-5p, miR-146a-5p and miR-423-5p.

A few studies examined the expression of miRNAs in human liver cancer cell line during DENV infection^12,13^ and only one in human patients during DHF^17^. This is the first study to describe the association of miR-126-5p in infectious diseases and the first on describing the hepatic miRNA profile in dengue (in vivo).

The majority of the miRNAs that were found differentially expressed in this study were downregulated in DHF. This was expected, since DENV can inhibit the expression levels of Dicer, Drosha, Ago1 and Ago2, key players in the miRNA biogenesis, resulting in the suppression several miRNAs^12^. Kakumani et al*.* detected the downregulation of 143 miRNAs and the overexpression of only 3 miRNAs. They could validate the suppression of some miRNAs by qRT-PCR during DENV infection, including miR-10b-5p, also found to be downregulated in the present study.

Kanokudom et al. also studied the regulation of selected miRNAs during DENV infection in HepG2 cells, and observed the downregulation of miR-146a^13^. Surprisingly, the miR-146a-5p is usually up-regulated in dengue fever, targeting the inflammatory signaling pathway to inhibit type I IFN-mediated antiviral defense by regulating NF-kb. It’s up-regulation might serve as a mechanism of DENV to establish its initial infection and later dissemination^14^. Also, is well established that autophagy, a cellular degradation and recycling process^15^, is required for efficient DENV replication^16^. Pu et al. found a negative correlation between the levels of miR-146a and DENV-2 induced autophagy by targeting TRAF6, suggesting that this is necessary in order to suppress excessive inflammation and reduce pathological damage^16^. The downregulation of miR-146a in DHF, detected in this study, could be increasing DENV-2 autophagy and consequently promoting the inflammation response and pathological damage.

Only one study assessed the expression of miRNAs in patients with DHF^17^. In that study, the authors used mononuclear leukocytes derived from patients with and without DHF to evaluate eleven miRNAs that targets SOCS1, which in turn is a negative regulator of several cytokines involved in the immune response^18^. SOCS1 acts by inhibiting JAK, an activator of a family of transcription factors known as STATs that induces the transcription of several cytokines^18^. In the present study, miR-204-5p, previously described as a JAK2 inhibitor^19^, was found to be downregulated and miR-126, which activates the JAK1/STAT3 pathway by inhibiting ZEB1^20^ was found to be upregulated. Taken together, these findings suggest that the tight regulation of cytokines by SOCS-1 and JAKs/STATs cascade might have a direct impact in the development of DHF.

The miR-126-5p is located in the intronic *Egfl7* gene. This region encodes the guide sequence miRNA-126-3p (named miR-126) and the passenger 5p. Both *Egfl7* gene and miR-126-3p/5p are angiogenic factors involved in maintaining vascular integrity^21^. The miR-126-3p had one differentially expressed isomiR and the miR-126-5p had five, all up regulated in DHF. The growth and endothelial repair regulation by miR-126-5p (and miR-126-3p) are well established in literature, as well as the vascular permeability increase in dengue hemorrhagic pathogenesis, in this way is evident this possible relationship between miR-126-5p super-expression and DHF vascular symptoms^22^. It is possible that vascular permeability in DHF be regulated by miR-126-5p, however, due the lack of data related to gene expression and cytokine profile from samples in this study, further experimental analysis to elucidate the real mechanism that describe this association are necessary.

The miRNA miR-122-5p was the most expressed miRNA in the samples showing 28 differentially expressed isomiRs, 26 down-regulated in DHF group (as the canonical sequence) and two up-regulated. The miR-122-5p is a liver-specific miRNA that represents more than 50% of liver miRnome and plays important role in fat metabolism and homeostasis. Rats with this gene silenced developed fatty liver hepatitis, fibrosis and hepatocellular carcinoma^23^, that agrees with histopatologic results of steatosis, necrosis, hyperplasia and metabolic changes from DHF fatal cases^8^. The up-regulated isomiRs, were found in high levels in the DHF liver samples and in very low level in the control samples, this indicates that those isomiRs can play a different role in this condition, likely related to inflammation balancing acting towards reducing the production of vasoactive cytokines.

The enrichment analysis result of KEGG pathways from down regulated miRNA target genes in dengue (miR-122-5p, miR-10b-5p, miR-204-5p, miR-148a-5p, miR-146a-5p and miR-423-5p) described 29 pathways associated with cell death regulation, from which ten were found among the 30 best scoring pathways. This indicate a possible role of these miRNAs on DHF, once apoptosis is one of the main characteristics of dengue hemorrhagic pathogenesis^4^.

One sub-expressed, miR-148a-5p, was associated with cancer, and can also be related to cell proliferation control pathways^25^, but were never described in association to dengue pathogenesis as miR-126-5p, miR122-5p and miR146a-5p, an thus its role remains to be determined. Nevertheless, it showed statistically significant differences even with stringent analysis criteria and deserve further studies to clarify this relationship.

Of note, some miRNA present divergent expression pattern when compared to their respective isomiRs, suggesting that those divergent isomiRs might act differently from their canonical miRNA in these conditions. This analysis demonstrates that those expression results can be obscured by a DE analysis focusing only on total miRNA or canonical miRNA.

## Conclusion

Our study provided insight into the hepatic miRNA and isomiR profile in DHF, made a deep differential expression analysis, characterized the isomiRs representation for previously miRNA associated with the disease, and among the eight miRNAs differentially expressed in DHF human liver. We highlight miRNAs miR-126-5p, miR-122-5p and miR-146a-5p, which seems to be involved in liver pathogenesis of DHF, because the revised function in growth and endothelial repair linked to dengue due the vascular permeability presented in bleeding cases. The central role of miR-122-5p in liver homeostasis and interferon regulation of miR-146a-5p associated to dengue by its immune response involvement.

MiRNAs and isomiRNAs are promising agents for anti-viral therapy and diagnostics development, experimental studies are necessary to identify target genes regulated by these miRNAs in liver during DENV infection, also isomiRs discovery may alter the current knowledge of immune modulation that leads to severe dengue and fatal cases.

## Material and methods

This research study has been approved by the ethics committee of the Medical School of the University of São Paulo, Brazil (local number 253/12 CEP/FMUSP 08/2012). All methods were performed in accordance with the relevant guidelines and regulations of the University of São Paulo and Instituto Evandro Chagas.

### Samples

All procedures were carried out with the informed written consent of the next of kin. Liver tissues samples (n = 6) from patients who died from DHF during a DENV-2 outbreak in São Paulo State in 2010 were provided by the Department of Pathology from the Guilherme Álvaro Hospital (São Paulo, Brazil). Tissue samples were obtained by performing a viscerotomy within 12 h following death of patients and preserved using formalin-fixation and paraffin-embedding (FFPE). DENV infection was confirmed by clinical, serological (IgM and NS1 antigen detection by ELISA) and histological examination (hematoxylin–eosin staining detection of viral antigens by immunohistochemistry assay). Control FFPE liver tissue samples (n = 5) were obtained from patients that died of acute myocardial infarction. Histological analysis (performed within 12 h following death) showed no infection or hepatic injury (data not available).

### RNA extraction, quantification and sequencing

Small RNAs were extracted from 5 μm sections of FFPE liver tissue, using the High Pure miRNA Isolation Kit (Roche 454 Life Sciences, Branford, CT, USA) according to the manufacturer's protocol. Qubit 2.0 Fluorometer (Thermo Scientific, Waltham, MA, USA) and Small RNA Analysis kit in Agilent 2100 Bioanalyzer (Agilent Technologies, Santa Clara, CA, USA) were used to check the quality and quantity, respectively, of the extracted RNA.

Samples were sequenced at the Functional Genomics Center (Agriculture College Luiz de Queiroz—USP, Piracicaba, São Paulo) using Illumina MiSeq platform for small RNAs, according to the manufacturer's protocol. For library construction, the TruSeq Small RNA Library Prep (Illumina Inc., San Diego, CA, USA) kit was used, with reverse transcription by PCR reaction to synthesize the cDNA. Library fragments were selected in PAGE 6% and its quality accessed using Agilent 2100 Bioanalyzer.

### Data processing

Quality control of the raw reads was performed using FastQC (https://www.bioinformatics.babraham.ac.uk/projects/fastqc) and FASTX-toolkit (https://hannonlab.cshl.edu/fastx_toolkit/index.html) was used to trim 3′ adapters, remove unresolved bases (N) and eliminate reads shorter than 17nt. Collapsing of redundant sequences and mapping against the human genome (version hg19—UCSC) was performed by the Mapper module available in miRDeep2 software^27^. miRDeep2 was also used to calculate read counts and to annotate the mature miRNA. By the end of the processing step, only miRNAs with 18 to 26nt length were selected. The SeqBuster-mirAligner v.1.2.2^28^ allowed the identification of isomiRs by performing a survey on miRNA sequence variability in the samples, applying default parameters.

### Differential expression analysis

To determine the miRNAs expression levels, the miRNAs counts obtained by miRDeep2 were normalized using Counts per million (CPM). The differential expression analysis was performed with the edgeR package for R Bioconductor^29^. Exact Fisher and GLM (General Linear Model) statistical tests were applied with FDR (False Discovery Rate Benjamini-Hochberg) to adjust the p-value. True differential expressed miRNA was defined by a log2-transformed fold change (log2FC) ≥ 2 (or ≤ -2) and an FDR value < 0.05.

### Prediction of target genes and functional analysis

TargetScan (www.targetscan.org), miRDB (www.miRDB.org) and miRTarBase (www.miRTarBase.mbc.nctu.edu.tw) databases were used to predict the targets of the differentially expressed miRNA. Only targets predicted by at least two of the mentioned databases considered valid and proceeded to functional annotation and enrichment analysis with DAVID 6.7 (https://david.ncifcrf.gov/home.jsp)30. Only functional categories containing pathways with p-value (with Bejamini correction) < 0.05 were evaluated (Additional File 4). The interaction network of target genes and pathways was built with Cytoscape 3.3 platform (https://www.cytoscape.org/).

## Supplementary information

Supplementary file1

## References

[1] Bhatt S (2013). The global distribution and burden of dengue. Nature.

[2] Brady OJ (2012). Refining the global spatial limits of dengue virus transmission by evidence-based consensus. PLoS Negl. Trop. Dis..

[3] Salles TS (2018). History, epidemiology and diagnostics of dengue in the American and Brazilian contexts: a review. Parasit. Vectors.

[4] Islam R (2015). Dengue epidemiology and pathogenesis: images of the future viewed through a mirror of the past. Virol. Sin..

[5] Saúde, S. de V. em. Boletim Epidemiológico. Monitoramento dos casos de arboviroses humanas transmitidas pelo Aedes aegypti (dengue, chikungunya e zika), semanas epidemiológicas 1 a 32 de 2020. *Ministério da Saúde* **51**, 11–17 (2020).

[6] Sreekanth GP (2016). SB203580 modulates p38 MAPK signaling and Dengue virus-induced liver injury by reducing MAPKAPK2, HSP27, and ATF2 phosphorylation. PLoS ONE.

[7] Pagliari C (2014). Immunopathogenesis of Dengue Hemorrhagic Fever: Contribution to the Study of Human Liver Lesions. J. Med. Virol..

[8] Póvoa TF (2014). The pathology of severe dengue in multiple organs of human fatal cases: histopathology, ultrastructure and virus replication. PLoS ONE.

[9] John DV, Lin YS, Perng GC (2015). Biomarkers of severe dengue disease: a review. J. Biomed. Sci..

[10] Dogini DB (2014). The new world of RNAs. Genet. Mol. Biol..

[11] Tan GC, Dibb N (2015). IsomiRs have functional importance. Malays. J. Pathol..

[12] Kakumani PK (2013). Role of RNA interference (RNAi) in dengue virus replication and identification of NS4B as an RNAi suppressor. J. Virol..

[13] Kanokudom S (2017). miR-21 promotes dengue virus serotype 2 replication in HepG2 cells. Antiviral Res..

[14] Wu S (2013). MiR-146a facilitates replication of dengue virus by dampening interferon induction by targeting TRAF6. J. Infect..

[15] Yang Z, Klionsky DJ (2009). An overview of the molecular mechanism of Autophagy Zhifen. Curr. Top. Microbiol. Immunol..

[16] Pu J (2017). miR-146a Inhibits dengue-virus-induced autophagy by targeting TRAF6. Arch. Virol..

[17] Chen RF (2014). Augmented miR-150 expression associated with depressed SOCS1 expression involved in dengue haemorrhagic fever. J. Infect..

[18] Liau NPD (2018). The molecular basis of JAK/STAT inhibition by SOCS1. Nat. Commun..

[19] Wang X (2015). MicroRNA-204 targets JAK2 in breast cancer and induces cell apoptosis through the STAT3/BCl-2/survivin pathway. Int. J. Clin. Exp. Pathol..

[20] Jiang R, Zhang C, Liu G, Gu R, Wu H (2017). MicroRNA-126 inhibits proliferation, migration, invasion, and EMT in osteosarcoma by targeting ZEB1. J. Cell. Biochem..

[21] Nikolic I, Plate KH, Schmidt MHH (2010). EGFL7 meets miRNA-126: an angiogenesis alliance. J. Angiogenes. Res..

[22] Schober A (2014). MicroRNA-126-5p promotes endothelial proliferation and limits atherosclerosis by suppressing Dlk1. Nat. Med..

[23] Tsai WC (2012). MicroRNA-122 plays a critical role in liver homeostasis and hepatocarcinogenesis. J. Clin. Invest..

[24] Ouyang X (2016). Dysregulated serum miRNA profile and promising biomarkers in dengue-infected patients. Int. J. Med. Sci..

[25] Long XR, He Y, Huang C, Li J (2014). MicroRNA-148a is silenced by hypermethylation and interacts with DNA methyltransferase 1 in hepatocellular carcinogenesis. Int. J. Oncol..

[26] Miyamoto S (2015). Expression patterns of miRNA-423-5p in the serum and pericardial fluid in patients undergoing cardiac surgery. PLoS ONE.

[27] Friedländer MR, MacKowiak SD, Li N, Chen W, Rajewsky N (2012). MiRDeep2 accurately identifies known and hundreds of novel microRNA genes in seven animal clades. Nucleic Acids Res..

[28] Pantano L, Estivill X, Martí E (2009). SeqBuster, a bioinformatic tool for the processing and analysis of small RNAs datasets, reveals ubiquitous miRNA modifications in human embryonic cells. Nucleic Acids Res..

[29] Robinson MD, McCarthy DJ, Smyth GK (2009). edgeR: A Bioconductor package for differential expression analysis of digital gene expression data. Bioinformatics.

[30] Huang DW, Sherman BT, Lempicki RA (2009). Systematic and integrative analysis of large gene lists using DAVID bioinformatics resources. Nat. Protoc..

