# Supplementary material for: Differential expression analysis and profiling of hepatic miRNA and isomiRNA in dengue hemorrhagic fever
Source: Sci Rep. 2021 Mar 10;11:5554. doi: 10.1038/s41598-020-72892-w (PMC7946910; doi:10.1038/s41598-020-72892-w)
Supplement: Supplementary file 1 — Supplementary file1 [file 41598_2020_72892_MOESM1_ESM.docx]

**SUPPLEMENTARY MATERIAL**

**Title**

Differential expression analysis and profiling of hepatic miRNA and isomiRNA in dengue hemorrhagic fever

Layanna Freitas de Oliveira^1,5^*, Amanda Araújo Serrão de Andrade¹, Carla Pagliari^2^, Leda Viegas de Carvalho^3^, Taiana S. Silveira^4^ , Jedson Ferreira Cardoso^1^ , André Luiz Teles e Silva^5^ , Janaina Vasconcelos^1^ , Caroline Aquino Moreira-Nunes^6^, Rommel Burbano^5^, Márcio Nunes^1^ , Eduardo José Melo dos Santos^5^, João Lídio da Silva Gonçalves Vianez Júnior^1^*

1 Center for Technological Innovation, Instituto Evandro Chagas, Ananindeua, PA, Brazil.

2 Departamento de Patologia, Faculdade de Medicina da Universidade de São Paulo, São Paulo, SP, Brazil.

3 Fundação Oncocentro de São Paulo, São Paulo, SP, Brazil.

4 Faculdade de Medicina de São José do Rio Preto, São Paulo, SP, Brazil.

5 Instituto de Ciências Biológicas, Universidade Federal do Pará, Belém, PA, Brazil.

6 Laboratory of Pharmacogenetics, Drug Research and Development Center (NPDM), Universidade Federal do Ceará, Fortaleza, CE, Brazil.

* Corresponding authors

E-mail: layannafoliveira@gmail.com, vianez.iec@gmail.com

**
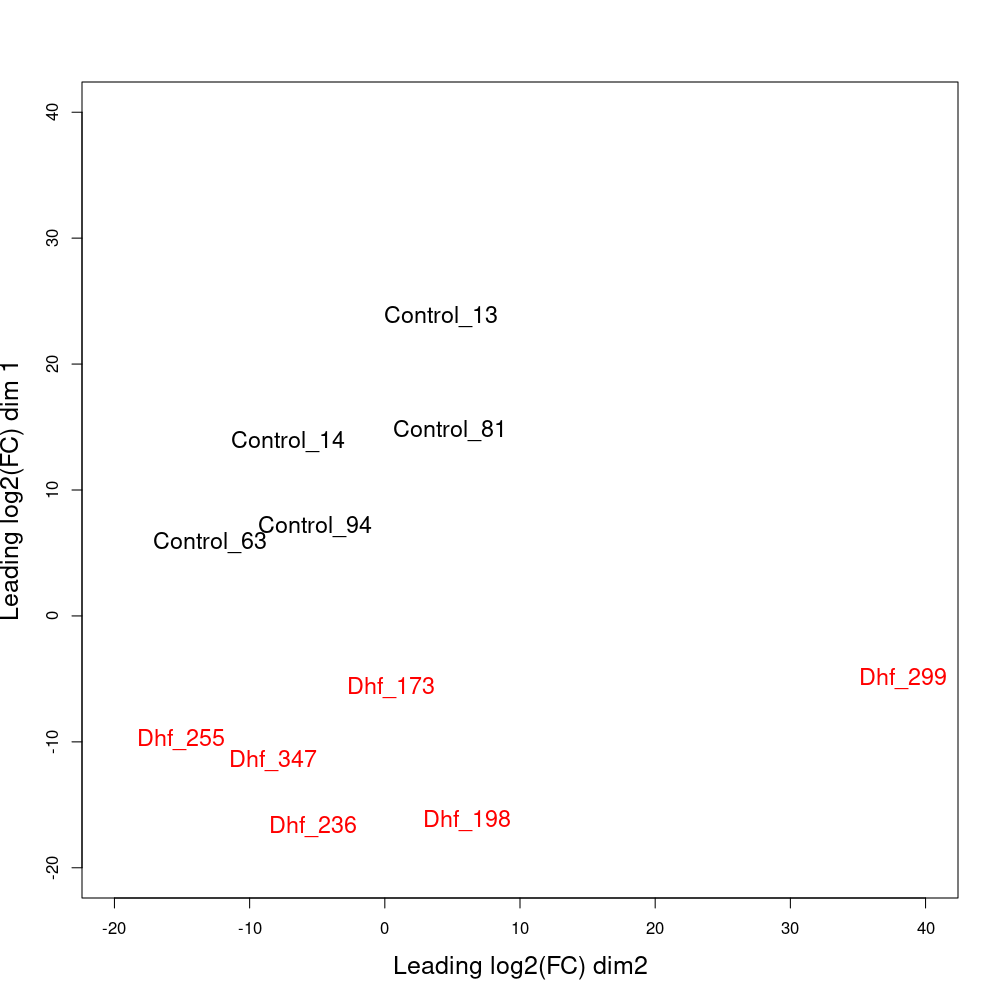
**

**Supplementary figure S1.** MDSPlot using log2(FC) shows a clear clustering of dengue hemorrhagic fever (DHF) and controls groups, with exception of sample Dhf_299 (excluded as outlier).

| miRNA ID | logFC | logCPM | GLM Test | |  | Controls (CPM expression) | | | | |  | Dengue Hemorrhagic Fever (CPM expression) | | | | |
| --- | --- | --- | --- | --- | --- | --- | --- | --- | --- | --- | --- | --- | --- | --- | --- | --- |
|  |  |  | PValue | FDR |  | Control  63 | Control  81 | Control  94 | Control  13 | Control  14 |  | Dhf_173 | Dhf_198 | Dhf_236 | Dhf_255 | Dhf_347 |
| **hsa-miR-133a-3p** | **6.7767** | **11.6553** | **0.00000000** | **0.00000000** |  | **43.72** | **118.21** | **54.42** | **55.28** | **18.91** |  | **16.51** | **48.33** | **96.63** | **31503.26** | **95.24** |
| **hsa-miR-122-5p** | **-6.5937** | **16.7992** | **0.00000000** | **0.00000000** |  | **105405.02** | **10267.63** | **15628.35** | **726378.27** | **271308.42** |  | **4568.23** | **10.36** | **1269.06** | **2150.65** | **3685.83** |
| **hsa-miR-10b-5p** | **-6.1663** | **16.0610** | **0.00000000** | **0.00000001** |  | **650029.31** | **5401.20** | **2727.72** | **10745.30** | **5262.59** |  | **1934.44** | **2651.49** | **1518.15** | **1792.91** | **1489.84** |
| **hsa-miR-204-5p** | **-5.6317** | **10.4449** | **0.00000000** | **0.00000013** |  | **12761.74** | **208.28** | **161.09** | **160.83** | **272.59** |  | **61.53** | **31.07** | **49.39** | **70.70** | **58.51** |
| **hsa-miR-126-5p** | **3.0933** | **13.6270** | **0.00007561** | **0.00675425** |  | **4103.75** | **903.48** | **3657.28** | **713.67** | **3874.07** |  | **24969.17** | **13896.14** | **34649.08** | **17659.07** | **21976.20** |
| **hsa-miR-148a-5p** | **-3.1354** | **9.3225** | **0.00008324** | **0.00721739** |  | **201.48** | **1548.03** | **766.29** | **1140.87** | **2082.79** |  | **184.59** | **96.67** | **64.42** | **192.30** | **112.93** |
| **hsa-miR-146a-5p** | **-2.6476** | **12.2746** | **0.00057794** | **0.03872188** |  | **5139.67** | **1415.74** | **803.30** | **14655.42** | **20727.72** |  | **1260.61** | **2064.57** | **1007.09** | **1529.92** | **959.21** |
| **hsa-miR-423-5p** | **-2.7806** | **7.2592** | **0.00070059** | **0.04589619** |  | **114.05** | **315.23** | **450.63** | **306.58** | **169.12** |  | **46.52** | **37.98** | **10.74** | **55.14** | **44.90** |
| hsa-miR-223-3p | 2.5945 | 7.4584 | 0.00134798 | 0.07497797 |  | 38.02 | 19.70 | 84.90 | 25.13 | 70.09 |  | 415.70 | 307.27 | 446.64 | 134.33 | 156.47 |
| hsa-miR-142-3p | 2.3451 | 9.8276 | 0.00226425 | 0.10942615 |  | 167.27 | 481.30 | 182.86 | 291.50 | 369.38 |  | 1437.70 | 3162.45 | 1479.50 | 695.67 | 813.63 |
| hsa-miR-338-3p | 2.3752 | 8.1973 | 0.00257502 | 0.12243787 |  | 205.28 | 64.74 | 41.36 | 80.41 | 70.09 |  | 267.13 | 324.53 | 1056.48 | 257.34 | 500.70 |
| hsa-miR-193b-3p | -2.3916 | 6.9823 | 0.00332657 | 0.14002844 |  | 55.12 | 543.22 | 315.66 | 50.26 | 90.12 |  | 49.52 | 24.17 | 19.33 | 56.56 | 48.98 |
| hsa-miR-378a-5p | -2.5167 | 5.9155 | 0.00337246 | 0.14002844 |  | 53.22 | 92.88 | 84.90 | 45.23 | 186.92 |  | 16.51 | 13.81 | 6.44 | 19.80 | 23.13 |
| hsa-miR-144-5p | 2.1186 | 9.4112 | 0.00554004 | 0.21489512 |  | 606.34 | 53.48 | 422.33 | 100.52 | 87.90 |  | 313.65 | 2606.61 | 1294.83 | 709.81 | 613.62 |
| hsa-miR-15a-5p | 2.0464 | 9.4565 | 0.00716388 | 0.26733075 |  | 389.66 | 70.36 | 468.05 | 75.39 | 338.23 |  | 1134.55 | 417.75 | 1533.18 | 957.26 | 1519.78 |
| hsa-miR-320a | -1.9509 | 8.9860 | 0.01069315 | 0.37222214 |  | 243.30 | 1967.40 | 1179.91 | 532.74 | 130.17 |  | 348.17 | 283.10 | 94.48 | 131.50 | 190.48 |
| hsa-miR-7641 | 2.0813 | 6.4534 | 0.01161616 | 0.37222214 |  | 100.74 | 8.44 | 6.53 | 15.08 | 21.14 |  | 219.11 | 138.10 | 88.04 | 69.28 | 142.86 |
| hsa-miR-146b-5p | -1.8673 | 14.0306 | 0.01222926 | 0.38100765 |  | 12121.18 | 5533.49 | 3574.56 | 34839.30 | 75259.55 |  | 5240.55 | 12781.00 | 6081.20 | 5756.27 | 6137.61 |
| hsa-miR-500a-3p | -2.0466 | 6.3821 | 0.01309387 | 0.39084339 |  | 239.50 | 84.44 | 95.79 | 80.41 | 136.85 |  | 16.51 | 34.52 | 32.21 | 38.18 | 32.65 |
| hsa-miR-218-5p | 1.9802 | 6.3473 | 0.01670700 | 0.42828028 |  | 66.53 | 28.15 | 17.42 | 30.16 | 18.91 |  | 25.51 | 248.58 | 212.58 | 63.63 | 92.52 |
| hsa-miR-199b-5p | 1.8547 | 7.9577 | 0.01706409 | 0.43366336 |  | 125.45 | 140.73 | 71.84 | 145.75 | 50.07 |  | 132.06 | 583.47 | 418.73 | 329.46 | 462.60 |
| hsa-miR-218-5p | 1.9670 | 6.3365 | 0.01744434 | 0.43581286 |  | 66.53 | 28.15 | 17.42 | 30.16 | 18.91 |  | 24.01 | 248.58 | 208.29 | 63.63 | 92.52 |
| hsa-miR-374b-5p | -1.8475 | 7.6385 | 0.01788233 | 0.43930932 |  | 395.36 | 36.59 | 100.14 | 165.85 | 778.82 |  | 133.57 | 55.24 | 49.39 | 73.53 | 97.96 |
| hsa-miR-30c-2-3p | -1.9744 | 5.9885 | 0.01870760 | 0.45578510 |  | 159.66 | 112.58 | 39.19 | 135.70 | 63.42 |  | 19.51 | 55.24 | 12.88 | 26.87 | 16.33 |
| hsa-miR-10a-5p | -1.6986 | 15.7999 | 0.02199444 | 0.50655939 |  | 192902.94 | 40437.23 | 44867.03 | 75056.27 | 82829.67 |  | 35304.70 | 32722.39 | 32871.11 | 13728.24 | 19727.15 |
| hsa-miR-148a-3p | -1.6238 | 14.3892 | 0.02815700 | 0.58108723 |  | 8283.53 | 10912.17 | 42391.83 | 16268.73 | 84055.76 |  | 14728.18 | 2585.89 | 4713.36 | 19419.47 | 11087.42 |
| hsa-miR-409-3p | -1.7391 | 6.6107 | 0.03149254 | 0.62308739 |  | 15.21 | 298.35 | 176.33 | 155.80 | 114.60 |  | 21.01 | 65.60 | 32.21 | 60.80 | 47.62 |
| hsa-miR-335-5p | -1.7118 | 6.8524 | 0.03215285 | 0.63191072 |  | 106.44 | 14.07 | 84.90 | 145.75 | 470.63 |  | 61.53 | 41.43 | 42.95 | 35.35 | 69.39 |
| hsa-miR-378a-3p | -1.5799 | 12.5734 | 0.03278822 | 0.64001420 |  | 3371.96 | 16625.79 | 11557.45 | 7106.57 | 7000.47 |  | 2582.76 | 897.64 | 1090.84 | 7994.59 | 2704.85 |
| hsa-miR-144-3p | 1.5483 | 10.8107 | 0.03749770 | 0.70409695 |  | 3138.16 | 106.95 | 659.62 | 417.15 | 248.11 |  | 697.84 | 6128.11 | 2890.29 | 1716.56 | 1948.36 |
| hsa-miR-374a-5p | -1.5795 | 8.2924 | 0.03871920 | 0.70897023 |  | 406.76 | 39.40 | 71.84 | 392.02 | 1369.61 |  | 186.09 | 179.53 | 130.99 | 125.84 | 141.50 |
| hsa-miR-141-3p | 1.5114 | 11.3514 | 0.04163799 | 0.74141550 |  | 4662.58 | 244.87 | 400.56 | 1065.48 | 392.75 |  | 397.69 | 3804.61 | 6723.25 | 2270.84 | 6100.87 |
| hsa-miR-98-5p | -1.5176 | 9.6444 | 0.04225163 | 0.74141550 |  | 1366.65 | 166.06 | 574.72 | 1015.22 | 2748.12 |  | 666.32 | 345.25 | 304.92 | 446.81 | 287.08 |
| hsa-let-7f-2-3p | -1.7226 | 5.0514 | 0.04197370 | 0.74141550 |  | 38.02 | 25.33 | 39.19 | 60.31 | 65.64 |  | 21.01 | 6.90 | 8.59 | 18.38 | 12.25 |
| hsa-miR-92b-3p | 1.5835 | 7.1985 | 0.04409139 | 0.76012525 |  | 150.16 | 53.48 | 93.61 | 15.08 | 30.04 |  | 91.54 | 110.48 | 171.79 | 500.55 | 160.55 |
| hsa-miR-27a-3p | 1.4795 | 9.9809 | 0.04714703 | 0.76789743 |  | 657.66 | 185.76 | 711.86 | 246.27 | 840.01 |  | 1229.10 | 1491.46 | 1893.93 | 1600.61 | 1164.66 |
| hsa-miR-30a-5p | -1.4517 | 15.4442 | 0.04867298 | 0.78839536 |  | 199781.80 | 18477.79 | 20108.53 | 37050.68 | 51016.01 |  | 15208.41 | 33426.69 | 28655.93 | 22145.60 | 19906.74 |

**Supplementary table S1.** Resume of differential expression analysis, showing the first microRNAs with p-value < 0,05. Expression data are in counts per million.

**Supplementary Table S2.** Complete Annotation Clustering Analysis from DAVID 6.7 using the target genes of miRNA upregulated in dengue hemorrhagic fever (DHF) group. Enrichment Score > 1.3 and adjusted p-value ≤ 0.05 were considered relevant.

| **Via** | **Count** | **Genes** | **Benjamini** |
| --- | --- | --- | --- |
| Annotation Cluster 1 - Enrichment Score: 2,8987 |  |  |  |
| hsa05200:Pathways in cancer | 16 | EGFR, IL8, SMAD4, BRCA2, FADD, NFKB1, KIT, APPL1, CCDC6, IGF1R, CDKN1A, RAC1, RHOA, LAMC1, FAS, TRAF6 | 0,000027 |
| GO:0043067~regulation of programmed cell death | 19 | EGFR, IRAK1, ROCK1, TLR2, BRCA2, FADD, NFKB1, TLR4, KIT, BRCA1, IGF1R, CDKN1A, CD40LG, HIPK3, RAC1, RHOA, FAF1, FAS, TRAF6 | 0,035849 |
| GO:0010941~regulation of cell death | 19 | EGFR, IRAK1, ROCK1, TLR2, BRCA2, FADD, NFKB1, TLR4, KIT, BRCA1, IGF1R, CDKN1A, CD40LG, HIPK3, RAC1, RHOA, FAF1, FAS, TRAF6 | 0,018989 |
| GO:0043069~negative regulation of programmed cell death | 12 | EGFR, IGF1R, IRAK1, CDKN1A, ROCK1, CD40LG, HIPK3, RHOA, NFKB1, KIT, FAS, TRAF6 | 0,033021 |
| GO:0060548~negative regulation of cell death | 12 | EGFR, IGF1R, IRAK1, CDKN1A, ROCK1, CD40LG, HIPK3, RHOA, NFKB1, KIT, FAS, TRAF6 | 0,025489 |
| GO:0042981~regulation of apoptosis | 18 | EGFR, IRAK1, ROCK1, TLR2, BRCA2, FADD, NFKB1, TLR4, BRCA1, IGF1R, CDKN1A, CD40LG, HIPK3, RAC1, RHOA, FAF1, FAS, TRAF6 | 0,018847 |
| GO:0043066~negative regulation of apoptosis | 11 | EGFR, IGF1R, IRAK1, CDKN1A, ROCK1, CD40LG, HIPK3, RHOA, NFKB1, FAS, TRAF6 | 0,032020 |
| Annotation Cluster 2 - Enrichment Score: 2,45557 |  |  |  |
| GO:0010647~positive regulation of cell communication | 11 | EGFR, ERBB4, CDKN2AIP, RAC1, RHOA, SMAD4, FADD, TLR4, KIT, TRAF6, FLNA | 0,025717 |
| h_nfkbPathway:NF-kB Signaling Pathway | 5 | IRAK1, NFKB1, FADD, TLR4, TRAF6 | 0,125149 |
| GO:0009967~positive regulation of signal transduction | 9 | CDKN2AIP, RAC1, RHOA, SMAD4, FADD, TLR4, KIT, TRAF6, FLNA | 0,075479 |
| GO:0051223~regulation of protein transport | 6 | CD40LG, RHOA, SMAD4, FAF1, TRAF6, FLNA | 0,074189 |
| GO:0043123~positive regulation of I-kappaB kinase/NF-kappaB cascade | 5 | RHOA, FADD, TLR4, TRAF6, FLNA | 0,111252 |
| GO:0043122~regulation of I-kappaB kinase/NF-kappaB cascade | 5 | RHOA, FADD, TLR4, TRAF6, FLNA | 0,136083 |
| GO:0010740~positive regulation of protein kinase cascade | 5 | RHOA, FADD, TLR4, TRAF6, FLNA | 0,323119 |
| GO:0010627~regulation of protein kinase cascade | 6 | HIPK3, RHOA, FADD, TLR4, TRAF6, FLNA | 0,330829 |
| Annotation Cluster 3 - Enrichment Score: 2,349722 |  |  |  |
| IPR000488:Death | 5 | IRAK2, IRAK1, NFKB1, FADD, FAS | 0,020724 |
| SM00005:DEATH | 5 | IRAK2, IRAK1, NFKB1, FADD, FAS | 0,018212 |
| domain:Death | 4 | IRAK2, NFKB1, FADD, FAS | 0,189854 |
| Annotation Cluster 4 - Enrichment Score: 2,200633 |  |  |  |
| GO:0032880~regulation of protein localization | 8 | SEPT2, CD40LG, RHOA, SMAD4, FAF1, TRAF6, APPL1, FLNA | 0,021151 |
| GO:0070201~regulation of establishment of protein localization | 7 | CD40LG, RHOA, SMAD4, FAF1, TRAF6, APPL1, FLNA | 0,033177 |
| Annotation Cluster 5 - Enrichment Score: 2,089863 |  |  |  |
| GO:0031264~death-inducing signaling complex | 3 | FADD, FAF1, FAS | 0,098835 |
| Apoptosis | 8 | ROCK1, HIPK3, GZMB, NFKB1, FADD, FAF1, FAS, CCAR1 | 0,178631 |
| h_fasPathway:FAS signaling pathway ( CD95 ) | 3 | FADD, FAF1, FAS | 0,900178 |
| Annotation Cluster 6 - Enrichment Score: 2,086000 |  |  |  |
| hsa04620:Toll-like receptor signaling pathway | 8 | IRAK1, IL8, RAC1, TLR2, NFKB1, FADD, TLR4, TRAF6 | 0,002280 |
| GO:0007249~I-kappaB kinase/NF-kappaB cascade | 6 | IRAK2, IRAK1, TLR2, TLR4, FAF1, TRAF6 | 0,021038 |
| GO:0002224~toll-like receptor signaling pathway | 4 | IRAK2, IRAK1, TLR2, TLR4 | 0,024736 |
| GO:0002221~pattern recognition receptor signaling pathway | 4 | IRAK2, IRAK1, TLR2, TLR4 | 0,036166 |
| GO:0002218~activation of innate immune response | 4 | IRAK2, IRAK1, TLR2, TLR4 | 0,034030 |
| GO:0002758~innate immune response-activating signal transduction | 4 | IRAK2, IRAK1, TLR2, TLR4 | 0,034030 |
| GO:0007243~protein kinase cascade | 11 | EGFR, IRAK2, IGF1R, IRAK1, CXCR4, TLR2, TLR4, FAF1, KIT, TRAF6, SLC9A1 | 0,036842 |
| GO:0002757~immune response-activating signal transduction | 5 | IRAK2, IRAK1, TLR2, TLR4, TRAF6 | 0,046867 |
| GO:0002253~activation of immune response | 6 | IRAK2, IRAK1, CFH, TLR2, TLR4, TRAF6 | 0,047374 |
| Annotation Cluster 7 - Enrichment Score: 2,008796 |  |  |  |
| GO:0031981~nuclear lumen | 21 | SEPT2, MTA2, SMAD4, BRCA2, NFKB1, KIT, APPL1, SRF, BRCA1, PA2G4, BRPF1, PAPOLA, CDKN1A, CDKN2AIP, JAZF1, ZNF540, POU3F2, GTF3C2, CCNA2, NOVA1, MED1 | 0,134747 |
| GO:0070013~intracellular organelle lumen | 23 | SEPT2, MTA2, SMAD4, BRCA2, NFKB1, KIT, APPL1, SRF, BRCA1, PA2G4, BRPF1, PAPOLA, CDKN1A, P4HA1, PPM1K, CDKN2AIP, JAZF1, ZNF540, POU3F2, GTF3C2, CCNA2, NOVA1, MED1 | 0,218602 |
| GO:0043233~organelle lumen | 23 | SEPT2, MTA2, SMAD4, BRCA2, NFKB1, KIT, APPL1, SRF, BRCA1, PA2G4, BRPF1, PAPOLA, CDKN1A, P4HA1, PPM1K, CDKN2AIP, JAZF1, ZNF540, POU3F2, GTF3C2, CCNA2, NOVA1, MED1 | 0,228781 |
| GO:0031974~membrane-enclosed lumen | 23 | SEPT2, MTA2, SMAD4, BRCA2, NFKB1, KIT, APPL1, SRF, BRCA1, PA2G4, BRPF1, PAPOLA, CDKN1A, P4HA1, PPM1K, CDKN2AIP, JAZF1, ZNF540, POU3F2, GTF3C2, CCNA2, NOVA1, MED1 | 0,208620 |
| GO:0005654~nucleoplasm | 14 | MTA2, SMAD4, BRCA2, NFKB1, APPL1, BRCA1, BRPF1, CDKN1A, CDKN2AIP, JAZF1, POU3F2, GTF3C2, CCNA2, MED1 | 0,193918 |
| GO:0010628~positive regulation of gene expression | 11 | IRAK1, BRPF1, MTA2, SMAD4, NFKB1, POU3F2, KIT, SRF, CCNA2, BRCA1, MED1 | 0,159393 |
| GO:0045941~positive regulation of transcription | 10 | IRAK1, BRPF1, MTA2, SMAD4, NFKB1, POU3F2, SRF, CCNA2, BRCA1, MED1 | 0,263939 |
| GO:0005730~nucleolus | 9 | PAPOLA, PA2G4, SEPT2, MTA2, CDKN2AIP, ZNF540, KIT, SRF, NOVA1 | 0,608342 |
| Annotation Cluster 8 - Enrichment Score: 1,9924921 |  |  |  |
| hsa04620:Toll-like receptor signaling pathway | 8 | IRAK1, IL8, RAC1, TLR2, NFKB1, FADD, TLR4, TRAF6 | 0,002280 |
| h_nfkbPathway:NF-kB Signaling Pathway | 5 | IRAK1, NFKB1, FADD, TLR4, TRAF6 | 0,125149 |
| hsa04722:Neurotrophin signaling pathway | 6 | IRAK2, IRAK1, RAC1, RHOA, NFKB1, TRAF6 | 0,122624 |
| h_tollPathway:Toll-Like Receptor Pathway | 5 | IRAK1, TLR2, NFKB1, TLR4, TRAF6 | 0,274997 |
| 99,NF-kB_activation | 4 | IRAK1, NFKB1, FADD, TRAF6 | 0,343822 |
| 92,Ancient_Host_Defense_Pathways | 3 | IRAK1, NFKB1, TRAF6 | 0,394748 |
| h_RELAPathway:Acetylation and Deacetylation of RelA in The Nucleus | 3 | NFKB1, FADD, TRAF6 | 0,564379 |
| hsa04622:RIG-I-like receptor signaling pathway | 4 | IL8, NFKB1, FADD, TRAF6 | 0,207768 |
| hsa04621:NOD-like receptor signaling pathway | 3 | IL8, NFKB1, TRAF6 | 0,427530 |
| h_gsk3Pathway:Inactivation of Gsk3 by AKT | 3 | IRAK1, NFKB1, TLR4 | 0,900159 |
| Annotation Cluster 9 - Enrichment Score: 1,945966 |  |  |  |
| GO:0042802~identical protein binding | 15 | EGFR, ALDOA, IRAK2, IRAK1, ROCK1, SMAD4, FADD, NFKB1, SRF, FLNA, BRCA1, FOXP2, IGF1R, POU3F2, FAS | 0,054670 |
| GO:0046983~protein dimerization activity | 10 | EGFR, IRAK2, IRAK1, BACH2, SMAD4, NFKB1, SRF, FLNA, CREBL2, FOXP2 | 0,512785 |
| GO:0042803~protein homodimerization activity | 7 | IRAK2, IRAK1, SMAD4, NFKB1, SRF, FLNA, FOXP2 | 0,622882 |
| GO:0046982~protein heterodimerization activity | 4 | EGFR, IRAK2, IRAK1, FOXP2 | 0,884506 |
| Annotation Cluster 10 - Enrichment Score: 1,840182 |  |  |  |
| GO:0040012~regulation of locomotion | 8 | EGFR, IGF1R, ERBB4, IL8, CXCR4, CLIC4, RAC1, KIT | 0,046841 |
| GO:0042325~regulation of phosphorylation | 12 | EGFR, IRAK1, CDKN1A, CXCR4, HIPK3, RAC1, SMAD4, TLR4, KIT, TRAF6, CDKN3, CCNG1 | 0,049236 |
| GO:0051174~regulation of phosphorus metabolic process | 12 | EGFR, IRAK1, CDKN1A, CXCR4, HIPK3, RAC1, SMAD4, TLR4, KIT, TRAF6, CDKN3, CCNG1 | 0,054009 |
| GO:0019220~regulation of phosphate metabolic process | 12 | EGFR, IRAK1, CDKN1A, CXCR4, HIPK3, RAC1, SMAD4, TLR4, KIT, TRAF6, CDKN3, CCNG1 | 0,054009 |
| GO:0043549~regulation of kinase activity | 10 | EGFR, IRAK1, CDKN1A, CXCR4, HIPK3, RAC1, KIT, TRAF6, CDKN3, CCNG1 | 0,071427 |
| GO:0008283~cell proliferation | 11 | EGFR, PA2G4, ERBB4, CXCR4, CD40LG, NUMB, RAC1, BRCA2, POU3F2, KIT, APPL1 | 0,075369 |
| GO:0030334~regulation of cell migration | 7 | EGFR, IGF1R, ERBB4, CXCR4, CLIC4, RAC1, KIT | 0,068663 |
| GO:0051338~regulation of transferase activity | 10 | EGFR, IRAK1, CDKN1A, CXCR4, HIPK3, RAC1, KIT, TRAF6, CDKN3, CCNG1 | 0,070515 |
| GO:0051270~regulation of cell motion | 7 | EGFR, IGF1R, ERBB4, CXCR4, CLIC4, RAC1, KIT | 0,087436 |
| GO:0045859~regulation of protein kinase activity | 9 | EGFR, IRAK1, CDKN1A, CXCR4, HIPK3, KIT, TRAF6, CDKN3, CCNG1 | 0,103110 |
| kinase | 10 | EGFR, IGF1R, IRAK1, CDKN1A, ERBB4, ROCK1, HIPK3, KIT, CDKN3, PIP4K2A | 0,241484 |
| GO:0033674~positive regulation of kinase activity | 6 | EGFR, IRAK1, CXCR4, RAC1, KIT, TRAF6 | 0,287890 |
| GO:0044093~positive regulation of molecular function | 10 | EGFR, IRAK2, IRAK1, CXCR4, RAC1, TLR2, GNG13, TLR4, KIT, TRAF6 | 0,290031 |
| GO:0051347~positive regulation of transferase activity | 6 | EGFR, IRAK1, CXCR4, RAC1, KIT, TRAF6 | 0,310224 |
| hsa04144:Endocytosis | 6 | EGFR, IGF1R, ERBB4, CXCR4, KIT, TRAF6 | 0,201926 |
| GO:0045860~positive regulation of protein kinase activity | 5 | EGFR, IRAK1, CXCR4, KIT, TRAF6 | 0,521061 |
| GO:0043405~regulation of MAP kinase activity | 4 | EGFR, CXCR4, HIPK3, KIT | 0,527495 |
| GO:0006468~protein amino acid phosphorylation | 9 | EGFR, IRAK2, IGF1R, IRAK1, ERBB4, ROCK1, CXCR4, HIPK3, KIT | 0,619938 |
| GO:0043406~positive regulation of MAP kinase activity | 3 | EGFR, CXCR4, KIT | 0,719839 |
| GO:0043085~positive regulation of catalytic activity | 7 | EGFR, IRAK1, CXCR4, RAC1, GNG13, KIT, TRAF6 | 0,739688 |
| GO:0032147~activation of protein kinase activity | 3 | EGFR, IRAK1, TRAF6 | 0,768653 |
| GO:0048609~reproductive process in a multicellular organism | 5 | EGFR, CXCR4, BRCA2, KIT, STRBP | 0,965453 |
| GO:0032504~multicellular organism reproduction | 5 | EGFR, CXCR4, BRCA2, KIT, STRBP | 0,965453 |

**Supplementary Table S3.** Complete Annotation Clustering Analysis from DAVID 6.7 using the target genes of miRNA downregulated in dengue hemorrhagic fever (DHF) group. Enrichment Score > 1.3 and adjusted P value ≤ 0.05 were considered relevant

| **Term** | **Count** | **Genes** | **Benjamini** |
| --- | --- | --- | --- |
| Cluster 1 - Enrichment Score: 5,6477 |  |  |  |
| GO:0043067~regulation of programmed cell death | 60 | CADM1, TLR2, NFKB1, TLR4, ZFP91, BDNF, CDKN2A, TIAM1, RHOA, FAS, DAP, EGFR, ARHGEF4, IRAK1, ROCK1, ARHGEF18, TP53, FADD, ARHGEF12, BCL2L11, GRM4, NME2, CD40LG, IGF2R, HIPK3, FOXC1, NGFR, DCUN1D3, EIF2AK3, YWHAZ, BCLAF1, MCL1, PML, BCL2L2, NR3C1, KIT, CALR, SLC11A2, IGF1R, SOS1, BCL2, RB1CC1, RAC1, TGM2, TRAF6, PTCRA, NACC1, TGFBR1, NF1, MUL1, BRCA2, BIRC2, SIRT1, BRCA1, NOTCH2, SON, NOTCH1, CDKN1A, FAF1, DNM2 | 0,0000006 |
| GO:0010941~regulation of cell death | 60 | CADM1, TLR2, NFKB1, TLR4, ZFP91, BDNF, CDKN2A, TIAM1, RHOA, FAS, DAP, EGFR, ARHGEF4, IRAK1, ROCK1, ARHGEF18, TP53, FADD, ARHGEF12, BCL2L11, GRM4, NME2, CD40LG, IGF2R, HIPK3, FOXC1, NGFR, DCUN1D3, EIF2AK3, YWHAZ, BCLAF1, MCL1, PML, BCL2L2, NR3C1, KIT, CALR, SLC11A2, IGF1R, SOS1, BCL2, RB1CC1, RAC1, TGM2, TRAF6, PTCRA, NACC1, TGFBR1, NF1, MUL1, BRCA2, BIRC2, SIRT1, BRCA1, NOTCH2, SON, NOTCH1, CDKN1A, FAF1, DNM2 | 0,0000003 |
| GO:0042981~regulation of apoptosis | 59 | CADM1, TLR2, NFKB1, TLR4, ZFP91, BDNF, CDKN2A, TIAM1, RHOA, FAS, DAP, EGFR, ARHGEF4, IRAK1, ROCK1, ARHGEF18, TP53, FADD, ARHGEF12, BCL2L11, GRM4, NME2, CD40LG, IGF2R, HIPK3, FOXC1, NGFR, DCUN1D3, EIF2AK3, YWHAZ, BCLAF1, MCL1, PML, BCL2L2, NR3C1, CALR, SLC11A2, IGF1R, SOS1, BCL2, RB1CC1, RAC1, TGM2, TRAF6, PTCRA, NACC1, TGFBR1, NF1, MUL1, BRCA2, BIRC2, SIRT1, BRCA1, NOTCH2, SON, NOTCH1, CDKN1A, FAF1, DNM2 | 0,0000004 |
| GO:0043065~positive regulation of apoptosis | 34 | BCLAF1, CADM1, TLR2, PML, TLR4, NR3C1, CDKN2A, TIAM1, BCL2, SOS1, RAC1, TGM2, FAS, DAP, TRAF6, ARHGEF4, NACC1, TGFBR1, ARHGEF18, NF1, TP53, BRCA2, MUL1, FADD, ARHGEF12, BRCA1, BCL2L11, NOTCH2, CDKN1A, NOTCH1, FAF1, NGFR, DCUN1D3, DNM2 | 0,0004233 |
| GO:0043068~positive regulation of programmed cell death | 34 | BCLAF1, CADM1, TLR2, PML, TLR4, NR3C1, CDKN2A, TIAM1, BCL2, SOS1, RAC1, TGM2, FAS, DAP, TRAF6, ARHGEF4, NACC1, TGFBR1, ARHGEF18, NF1, TP53, BRCA2, MUL1, FADD, ARHGEF12, BRCA1, BCL2L11, NOTCH2, CDKN1A, NOTCH1, FAF1, NGFR, DCUN1D3, DNM2 | 0,0004031 |
| GO:0010942~positive regulation of cell death | 34 | BCLAF1, CADM1, TLR2, PML, TLR4, NR3C1, CDKN2A, TIAM1, BCL2, SOS1, RAC1, TGM2, FAS, DAP, TRAF6, ARHGEF4, NACC1, TGFBR1, ARHGEF18, NF1, TP53, BRCA2, MUL1, FADD, ARHGEF12, BRCA1, BCL2L11, NOTCH2, CDKN1A, NOTCH1, FAF1, NGFR, DCUN1D3, DNM2 | 0,0003417 |
| GO:0043069~negative regulation of programmed cell death | 29 | YWHAZ, MCL1, BCL2L2, NFKB1, KIT, ZFP91, IGF1R, BDNF, BCL2, RB1CC1, TGM2, RHOA, FAS, TRAF6, PTCRA, EGFR, IRAK1, ROCK1, TGFBR1, TP53, NOTCH2, NME2, CDKN1A, NOTCH1, SON, CD40LG, HIPK3, FOXC1, NGFR | 0,0007724 |
| GO:0060548~negative regulation of cell death | 29 | YWHAZ, MCL1, BCL2L2, NFKB1, KIT, ZFP91, IGF1R, BDNF, BCL2, RB1CC1, TGM2, RHOA, FAS, TRAF6, PTCRA, EGFR, IRAK1, ROCK1, TGFBR1, TP53, NOTCH2, NME2, CDKN1A, NOTCH1, SON, CD40LG, HIPK3, FOXC1, NGFR | 0,0007619 |
| GO:0043066~negative regulation of apoptosis | 28 | YWHAZ, MCL1, BCL2L2, NFKB1, ZFP91, IGF1R, BDNF, BCL2, RB1CC1, RHOA, TGM2, FAS, TRAF6, PTCRA, EGFR, IRAK1, ROCK1, TGFBR1, TP53, NOTCH2, NME2, CDKN1A, NOTCH1, SON, CD40LG, HIPK3, FOXC1, NGFR | 0,0011612 |
| GO:0006917~induction of apoptosis | 26 | ARHGEF4, NACC1, BCLAF1, CADM1, TGFBR1, ARHGEF18, TLR2, TP53, PML, MUL1, BRCA2, FADD, ARHGEF12, BRCA1, BCL2L11, NOTCH2, CDKN1A, CDKN2A, TIAM1, SOS1, RAC1, TGM2, NGFR, FAS, DAP, TRAF6 | 0,0013398 |
| GO:0012502~induction of programmed cell death | 26 | ARHGEF4, NACC1, BCLAF1, CADM1, TGFBR1, ARHGEF18, TLR2, TP53, PML, MUL1, BRCA2, FADD, ARHGEF12, BRCA1, BCL2L11, NOTCH2, CDKN1A, CDKN2A, TIAM1, SOS1, RAC1, TGM2, NGFR, FAS, DAP, TRAF6 | 0,0013083 |
| GO:0012501~programmed cell death | 38 | CADM1, MCL1, PML, BCL2L2, NFKB1, KIT, GREM1, SLC11A2, CDKN2A, CXCR4, TIAM1, SOS1, BCL2, RAC1, TGM2, FAS, DAP, TRAF6, CCAR1, ARHGEF4, ROCK1, ARHGEF18, KLF11, TP53, MUL1, GZMB, FADD, ARHGEF12, SIRT1, NTN1, BIRC2, BRCA1, BCL2L11, DNASE2, HIPK3, NGFR, FAF1, EIF2AK3 | 0,0022539 |
| GO:0008219~cell death | 42 | CADM1, MCL1, PML, BCL2L2, NFKB1, KIT, GREM1, SLC11A2, CDKN2A, CXCR4, TIAM1, SOS1, BCL2, RAC1, TGM2, FAS, DAP, TRAF6, CCAR1, ARHGEF4, ROCK1, ARHGEF18, KLF11, TP53, MUL1, GZMB, FADD, REEP1, ARHGEF12, SIRT1, BIRC2, NTN1, BRCA1, BCL2L11, ITPR1, DNASE2, EIF4G2, HIPK3, PLA2G6, NGFR, FAF1, EIF2AK3 | 0,0031491 |
| GO:0016265~death | 42 | CADM1, MCL1, PML, BCL2L2, NFKB1, KIT, GREM1, SLC11A2, CDKN2A, CXCR4, TIAM1, SOS1, BCL2, RAC1, TGM2, FAS, DAP, TRAF6, CCAR1, ARHGEF4, ROCK1, ARHGEF18, KLF11, TP53, MUL1, GZMB, FADD, REEP1, ARHGEF12, SIRT1, BIRC2, NTN1, BRCA1, BCL2L11, ITPR1, DNASE2, EIF4G2, HIPK3, PLA2G6, NGFR, FAF1, EIF2AK3 | 0,0035711 |
| GO:0006915~apoptosis | 36 | CADM1, MCL1, PML, BCL2L2, NFKB1, GREM1, SLC11A2, CDKN2A, CXCR4, TIAM1, SOS1, BCL2, RAC1, FAS, DAP, TRAF6, CCAR1, ARHGEF4, ROCK1, ARHGEF18, KLF11, TP53, MUL1, GZMB, FADD, ARHGEF12, SIRT1, NTN1, BIRC2, BRCA1, BCL2L11, DNASE2, HIPK3, NGFR, FAF1, EIF2AK3 | 0,0059353 |
| GO:0006916~anti-apoptosis | 18 | IRAK1, YWHAZ, MCL1, TGFBR1, BCL2L2, NFKB1, NOTCH2, IGF1R, BDNF, SON, CD40LG, HIPK3, BCL2, TGM2, FOXC1, FAS, NGFR, TRAF6 | 0,0063692 |
| Apoptosis | 23 | CADM1, ROCK1, MCL1, KLF11, TP53, MUL1, BCL2L2, FADD, NFKB1, GZMB, NTN1, SIRT1, BIRC2, BCL2L11, DNASE2, CDKN2A, HIPK3, BCL2, FAF1, DAP, FAS, NGFR, CCAR1 | 0,0176305 |
| Cluster 2 - Enrichment Score: 4,49715 |  |  |  |
| GO:0030528~transcription regulator activity | 80 | PPARA, BACH2, CDX2, THRB, ELF4, NR6A1, ZFP42, PAX6, NFKB1, DEK, ZEB1, RORA, ZNF253, CBFA2T3, HOXD10, BRPF1, CDKN2A, TBL1XR1, IRAK1, ZNF90, RREB1, MTA2, SOX12, TP53, UBE2I, SIX4, PPARGC1A, ELL2, FOXR2, PA2G4, NME2, VGLL3, FOXC1, PRDM2, TFAP2C, MED1, SUPT6H, BTAF1, BCLAF1, SCML2, PML, ZNF367, NR3C1, MYBL1, CALR, MYBL2, SRF, MEIS1, MEIS2, BCL2, HOXA10, POU2F1, HNRNPD, POU3F2, GTF3C2, ZNF423, CREBL2, NACC1, KLF12, KLF11, SMAD4, BRCA2, NR4A3, FOXP4, SIRT1, ATXN7L3, BRCA1, FOXP2, NOTCH2, NOTCH1, DMTF1, EBF2, SP4, JAZF1, ID4, NFIC, TCF12, NCOR2, KLF4, DNM2 | 0,0004872 |
| GO:0006357~regulation of transcription from RNA polymerase II promoter | 46 | PPARA, CDX2, THRB, ELF4, NR6A1, PAX6, ZNF367, NFKB1, DEK, RORA, ZEB1, CALR, SRF, HOXD10, MEIS2, POU2F1, RHOA, POU3F2, CHD3, TBL1XR1, KLF12, MTA2, SOX12, KLF11, TP53, SMAD4, SIX4, NR4A3, SNAI2, SIRT1, PPARGC1A, FOXP4, BRCA1, FOXP2, NOTCH1, SP4, JAZF1, FOXC1, ID4, TFAP2C, NFIC, TCF12, NCOR2, KLF4, MED1, SUPT6H | 0,0007192 |
| GO:0010558~negative regulation of macromolecule biosynthetic process | 37 | PPARA, BTAF1, BCLAF1, CDX2, THRB, NR6A1, PML, NFKB1, ZEB1, CALR, HIC2, MEIS2, POU2F1, TNRC6A, ZNF423, CDC6, TBL1XR1, NACC1, KLF12, MTA2, KLF11, SMAD4, TP53, BRCA2, UBE2I, SNAI2, SIRT1, FOXP4, BRCA1, FOXP2, PA2G4, JAZF1, ID4, NFIC, EIF2AK3, KLF4, NCOR2 | 0,0012428 |
| GO:0016481~negative regulation of transcription | 33 | PPARA, BTAF1, BCLAF1, CDX2, THRB, NR6A1, PML, NFKB1, ZEB1, CALR, HIC2, MEIS2, POU2F1, ZNF423, TBL1XR1, NACC1, KLF12, MTA2, KLF11, SMAD4, TP53, UBE2I, SNAI2, SIRT1, FOXP4, BRCA1, FOXP2, PA2G4, JAZF1, ID4, NFIC, KLF4, NCOR2 | 0,0011826 |
| GO:0010629~negative regulation of gene expression | 35 | PPARA, BTAF1, BCLAF1, CDX2, THRB, NR6A1, PML, NFKB1, ZEB1, CALR, HIC2, MEIS2, POU2F1, TNRC6A, ZNF423, TBL1XR1, NACC1, KLF12, MTA2, KLF11, SMAD4, TP53, UBE2I, SNAI2, SIRT1, FOXP4, BRCA1, FOXP2, PA2G4, JAZF1, ID4, CELF1, NFIC, KLF4, NCOR2 | 0,0011277 |
| GO:0045934~negative regulation of nucleobase, nucleoside, nucleotide and nucleic acid metabolic process | 35 | PPARA, BTAF1, BCLAF1, CDX2, THRB, NR6A1, PML, NFKB1, ZEB1, CALR, HIC2, MEIS2, POU2F1, ZNF423, CDC6, TBL1XR1, NACC1, KLF12, MTA2, KLF11, SMAD4, TP53, BRCA2, UBE2I, SNAI2, SIRT1, FOXP4, BRCA1, FOXP2, PA2G4, JAZF1, ID4, NFIC, KLF4, NCOR2 | 0,0013911 |
| GO:0031327~negative regulation of cellular biosynthetic process | 37 | PPARA, BTAF1, BCLAF1, CDX2, THRB, NR6A1, PML, NFKB1, ZEB1, CALR, HIC2, MEIS2, POU2F1, TNRC6A, ZNF423, CDC6, TBL1XR1, NACC1, KLF12, MTA2, KLF11, SMAD4, TP53, BRCA2, UBE2I, SNAI2, SIRT1, FOXP4, BRCA1, FOXP2, PA2G4, JAZF1, ID4, NFIC, EIF2AK3, KLF4, NCOR2 | 0,0013405 |
| GO:0051172~negative regulation of nitrogen compound metabolic process | 35 | PPARA, BTAF1, BCLAF1, CDX2, THRB, NR6A1, PML, NFKB1, ZEB1, CALR, HIC2, MEIS2, POU2F1, ZNF423, CDC6, TBL1XR1, NACC1, KLF12, MTA2, KLF11, SMAD4, TP53, BRCA2, UBE2I, SNAI2, SIRT1, FOXP4, BRCA1, FOXP2, PA2G4, JAZF1, ID4, NFIC, KLF4, NCOR2 | 0,0014846 |
| GO:0009890~negative regulation of biosynthetic process | 37 | PPARA, BTAF1, BCLAF1, CDX2, THRB, NR6A1, PML, NFKB1, ZEB1, CALR, HIC2, MEIS2, POU2F1, TNRC6A, ZNF423, CDC6, TBL1XR1, NACC1, KLF12, MTA2, KLF11, SMAD4, TP53, BRCA2, UBE2I, SNAI2, SIRT1, FOXP4, BRCA1, FOXP2, PA2G4, JAZF1, ID4, NFIC, EIF2AK3, KLF4, NCOR2 | 0,0017362 |
| GO:0010605~negative regulation of macromolecule metabolic process | 41 | BTAF1, PPARA, BCLAF1, CDX2, THRB, NR6A1, PML, NFKB1, FKBP1A, ZEB1, CALR, HIC2, MEIS2, CDKN2A, POU2F1, TNRC6A, ZNF423, CDC6, TBL1XR1, NACC1, KLF12, MTA2, KLF11, TP53, SMAD4, BRCA2, UBE2I, SNAI2, SIRT1, FOXP4, BRCA1, FLNA, FOXP2, PA2G4, JAZF1, CELF1, ID4, NFIC, EIF2AK3, KLF4, NCOR2 | 0,0071473 |
| GO:0045892~negative regulation of transcription, DNA-dependent | 25 | TBL1XR1, PPARA, CDX2, KLF12, MTA2, NR6A1, KLF11, TP53, SMAD4, NFKB1, UBE2I, ZEB1, SNAI2, CALR, SIRT1, FOXP4, FOXP2, HIC2, PA2G4, MEIS2, POU2F1, JAZF1, NFIC, NCOR2, KLF4 | 0,0071883 |
| GO:0051253~negative regulation of RNA metabolic process | 25 | TBL1XR1, PPARA, CDX2, KLF12, MTA2, NR6A1, KLF11, TP53, SMAD4, NFKB1, UBE2I, ZEB1, SNAI2, CALR, SIRT1, FOXP4, FOXP2, HIC2, PA2G4, MEIS2, POU2F1, JAZF1, NFIC, NCOR2, KLF4 | 0,0088477 |
| GO:0016564~transcription repressor activity | 23 | TBL1XR1, NACC1, BCLAF1, CDX2, KLF12, THRB, MTA2, PML, NFKB1, UBE2I, ZEB1, CALR, ZNF253, FOXP4, SIRT1, FOXP2, MEIS2, POU2F1, JAZF1, ID4, KLF4, NCOR2, ZNF423 | 0,0425488 |
| GO:0000122~negative regulation of transcription from RNA polymerase II promoter | 18 | PPARA, TBL1XR1, CDX2, KLF12, MTA2, NR6A1, KLF11, TP53, NFKB1, ZEB1, CALR, SNAI2, FOXP4, FOXP2, MEIS2, JAZF1, NFIC, NCOR2 | 0,0534878 |
| Cluster 3 - Enrichment Score: 4,451934 |  |  |  |
| activator | 39 | PPARA, BACH2, CDX2, ELF4, PML, ZNF367, NFKB1, MYBL1, CPEB1, ZEB1, RORA, SRF, MEIS1, BZW1, BRPF1, POU2F1, POU3F2, ZNF423, CREBL2, TBL1XR1, RREB1, KLF11, TP53, NCOA7, ZNF521, PPARGC1A, ATXN7L3, NOTCH2, NME2, NOTCH1, DMTF1, EBF2, SP4, PRDM2, CELF1, TFAP2C, NFIC, KLF4, MED1 | 0,0000031 |
| GO:0030528~transcription regulator activity | 80 | PPARA, BACH2, CDX2, THRB, ELF4, NR6A1, ZFP42, PAX6, NFKB1, DEK, ZEB1, RORA, ZNF253, CBFA2T3, HOXD10, BRPF1, CDKN2A, TBL1XR1, IRAK1, ZNF90, RREB1, MTA2, SOX12, TP53, UBE2I, SIX4, PPARGC1A, ELL2, FOXR2, PA2G4, NME2, VGLL3, FOXC1, PRDM2, TFAP2C, MED1, SUPT6H, BTAF1, BCLAF1, SCML2, PML, ZNF367, NR3C1, MYBL1, CALR, MYBL2, SRF, MEIS1, MEIS2, BCL2, HOXA10, POU2F1, HNRNPD, POU3F2, GTF3C2, ZNF423, CREBL2, NACC1, KLF12, KLF11, SMAD4, BRCA2, NR4A3, FOXP4, SIRT1, ATXN7L3, BRCA1, FOXP2, NOTCH2, NOTCH1, DMTF1, EBF2, SP4, JAZF1, ID4, NFIC, TCF12, NCOR2, KLF4, DNM2 | 0,0004872 |
| Transcription | 91 | PPARA, BACH2, CDX2, THRB, ELF4, NR6A1, ZFP42, NAA15, PAX6, BBX, NFKB1, ZEB1, RORA, ZNF253, CBFA2T3, HOXD10, BZW1, ZFP91, BRPF1, PCGF3, CDKN2A, PHTF2, ZNF540, CCAR1, TBL1XR1, KHDRBS3, ZNF90, RREB1, SOX12, TP53, ZNF649, NCOA7, PPARGC1A, ELL2, FOXR2, PA2G4, NME2, HIPK3, VGLL3, FOXC1, PRDM2, TFAP2C, MED1, SUV420H1, SUPT6H, BCLAF1, SCML2, PML, ZNF367, NR3C1, MYBL1, ZNF654, MYBL2, SRF, MEIS1, ZNF660, HIC2, RB1CC1, HOXA10, POU2F1, HNRNPD, POU3F2, GTF3C2, ZNF423, CHD3, CREBL2, SSRP1, NACC1, KLF12, WDR5, KLF11, SMAD4, ZNF521, NR4A3, SNAI2, FOXP4, SIRT1, ATXN7L3, FOXP2, NOTCH2, NOTCH1, BCORL1, DMTF1, EBF2, SP4, JAZF1, ZBTB2, NFIC, TCF12, NCOR2, KLF4 | 0,0001133 |
| transcription regulation | 89 | PPARA, BACH2, CDX2, THRB, ELF4, NR6A1, ZFP42, NAA15, PAX6, BBX, NFKB1, ZEB1, RORA, ZNF253, CBFA2T3, HOXD10, BZW1, ZFP91, BRPF1, PCGF3, CDKN2A, PHTF2, ZNF540, CCAR1, TBL1XR1, KHDRBS3, ZNF90, RREB1, SOX12, TP53, ZNF649, NCOA7, PPARGC1A, ELL2, FOXR2, PA2G4, NME2, HIPK3, VGLL3, FOXC1, PRDM2, TFAP2C, MED1, SUV420H1, SUPT6H, BCLAF1, SCML2, PML, ZNF367, NR3C1, MYBL1, ZNF654, MYBL2, SRF, ZNF660, HIC2, RB1CC1, HOXA10, POU2F1, HNRNPD, POU3F2, ZNF423, CHD3, CREBL2, SSRP1, NACC1, KLF12, WDR5, KLF11, SMAD4, ZNF521, NR4A3, SNAI2, FOXP4, SIRT1, ATXN7L3, FOXP2, NOTCH2, NOTCH1, BCORL1, DMTF1, EBF2, SP4, JAZF1, ZBTB2, NFIC, TCF12, NCOR2, KLF4 | 0,0001013 |
| GO:0006357~regulation of transcription from RNA polymerase II promoter | 46 | PPARA, CDX2, THRB, ELF4, NR6A1, PAX6, ZNF367, NFKB1, DEK, RORA, ZEB1, CALR, SRF, HOXD10, MEIS2, POU2F1, RHOA, POU3F2, CHD3, TBL1XR1, KLF12, MTA2, SOX12, KLF11, TP53, SMAD4, SIX4, NR4A3, SNAI2, SIRT1, PPARGC1A, FOXP4, BRCA1, FOXP2, NOTCH1, SP4, JAZF1, FOXC1, ID4, TFAP2C, NFIC, TCF12, NCOR2, KLF4, MED1, SUPT6H | 0,0007192 |
| GO:0031328~positive regulation of cellular biosynthetic process | 44 | PPARA, CDX2, THRB, ELF4, PAX6, NAA15, APOC2, TLR4, NFKB1, ZEB1, RORA, CALR, SRF, HOXD10, IGF1R, BRPF1, MEIS2, NPM2, POU2F1, POU3F2, SHC1, TRAF6, CCNA2, ZNF423, EGFR, IRAK1, TBL1XR1, KLF12, TGFBR1, MTA2, SMAD4, TP53, SIX4, NR4A3, PPARGC1A, BRCA1, ATXN7L3, NOTCH1, EBF2, FOXC1, NFIC, EIF5A2, KLF4, MED1 | 0,0007920 |
| GO:0009891~positive regulation of biosynthetic process | 44 | PPARA, CDX2, THRB, ELF4, PAX6, NAA15, APOC2, TLR4, NFKB1, ZEB1, RORA, CALR, SRF, HOXD10, IGF1R, BRPF1, MEIS2, NPM2, POU2F1, POU3F2, SHC1, TRAF6, CCNA2, ZNF423, EGFR, IRAK1, TBL1XR1, KLF12, TGFBR1, MTA2, SMAD4, TP53, SIX4, NR4A3, PPARGC1A, BRCA1, ATXN7L3, NOTCH1, EBF2, FOXC1, NFIC, EIF5A2, KLF4, MED1 | 0,0009189 |
| GO:0010557~positive regulation of macromolecule biosynthetic process | 42 | PPARA, CDX2, THRB, ELF4, PAX6, NAA15, TLR4, NFKB1, ZEB1, RORA, CALR, SRF, HOXD10, IGF1R, BRPF1, MEIS2, NPM2, POU2F1, POU3F2, SHC1, TRAF6, CCNA2, ZNF423, IRAK1, TBL1XR1, KLF12, TGFBR1, MTA2, SMAD4, TP53, SIX4, NR4A3, PPARGC1A, BRCA1, ATXN7L3, NOTCH1, EBF2, FOXC1, NFIC, EIF5A2, KLF4, MED1 | 0,0010586 |
| dna-binding | 81 | KIF22, PPARA, BACH2, CDX2, THRB, ELF4, NR6A1, ZFP42, PAX6, BBX, NFKB1, DEK, ZEB1, RORA, ZNF253, HOXD10, ZFP91, BRPF1, CDKN2A, PHTF2, ZNF540, ZNF90, RREB1, MTA2, SOX12, TP53, ZNF649, SIX4, TOX3, ZFR, HNRNPU, FOXR2, NME2, HIST2H2BE, FOXC1, PRDM2, TFAP2C, XRN1, MED1, BTAF1, BCLAF1, PML, ZNF367, NR3C1, MYBL1, ZNF654, MYBL2, SRF, MEIS1, ZNF660, HIC2, MEIS2, HOXA10, POU2F1, HNRNPD, POU3F2, STRBP, ZNF423, CHD3, CREBL2, SSRP1, SMG6, KLF12, RFX7, KLF11, ZNF521, NR4A3, SNAI2, FOXP4, SNAI1, BRCA1, FOXP2, SON, DMTF1, EBF2, SP4, ZBTB2, NFIC, TCF12, NCOR2, KLF4 | 0,0004779 |
| GO:0010604~positive regulation of macromolecule metabolic process | 50 | PPARA, CDX2, THRB, ELF4, PML, PAX6, NAA15, NFKB1, FKBP1A, TLR4, ZEB1, KIT, RORA, CALR, SRF, HOXD10, ZFP91, IGF1R, BRPF1, MEIS2, RB1CC1, BCL2, NPM2, POU2F1, POU3F2, FBXO4, SHC1, TRAF6, CCNA2, ZNF423, TBL1XR1, IRAK1, KLF12, MTA2, TGFBR1, TP53, SMAD4, NR4A3, SIX4, PPARGC1A, BRCA1, ATXN7L3, NOTCH1, EBF2, FOXC1, SH3D19, NFIC, EIF5A2, KLF4, MED1 | 0,0012039 |
| nucleus | 154 | SCAF1, CDX2, THRB, PNKD, BBX, NAA15, RBM6, INTS2, RORA, ZNF253, HOXD10, ZFP91, BRPF1, CDKN2A, PHTF2, CLK4, SPRED1, CCNA2, STAG2, RREB1, MTA2, ZNF649, SIX4, TOX3, PPARGC1A, CDKL2, HNRNPU, ELL2, NME2, PA2G4, CEP350, CAMK1, PRDM2, CELF1, VGLL3, TFAP2C, BIVM, FBXL3, SUPT6H, SUV420H1, MCL1, MYBL1, MYBL2, CCNG1, SRF, MEIS1, DAZAP1, FBXW7, MEIS2, NPM2, NUP50, HNRNPD, HOXA10, TRAF6, NOVA1, CREBL2, ZNF423, SMG6, KLF12, CLCC1, WDR5, RFX7, KLF11, SMAD4, ZNF521, NR4A3, FOXP4, RBMX, BRCA1, FOXP2, UBE2E3, NOTCH2, CDKN1A, NOTCH1, HNRNPH3, DMTF1, EBF2, CDKN2AIP, JAZF1, TCF12, KLF4, NCOR2, KIF22, PPARA, KDM6A, BACH2, ELF4, NR6A1, ZFP42, PAX6, DEK, NFKB1, ZEB1, CBFA2T3, CAMKK1, DIP2A, ZNF540, CDC6, TBL1XR1, KHDRBS3, ZNF90, SOX12, TP53, NCOA7, UBE2I, ZFR, RBBP6, FOXR2, HIST2H2BE, CLIC4, HIPK3, TPPP, FOXC1, EIF5A2, MED1, BTAF1, BCLAF1, FKBP5, SCML2, TRA2B, PML, ZNF367, NR3C1, ZNF654, ZNF660, SF3B1, HIC2, BCL2, RB1CC1, POU2F1, MSI1, POU3F2, GTF3C2, TNPO2, NMD3, CHD3, SSRP1, NACC1, APPL1, SNAI2, SNAI1, SIRT1, ATXN7L3, PAPOLA, SON, BCORL1, SP4, ZBTB2, ID4, SH3D19, FAF1, NFIC, DUSP8, RNF111 | 0,0007886 |
| GO:0051173~positive regulation of nitrogen compound metabolic process | 40 | PPARA, CDX2, THRB, ELF4, PAX6, NAA15, NFKB1, ZEB1, RORA, CALR, SRF, HOXD10, IGF1R, BRPF1, MEIS2, NPM2, POU2F1, POU3F2, SHC1, CCNA2, ZNF423, EGFR, IRAK1, TBL1XR1, KLF12, TGFBR1, MTA2, SMAD4, TP53, SIX4, NR4A3, PPARGC1A, BRCA1, ATXN7L3, NOTCH1, EBF2, FOXC1, NFIC, KLF4, MED1 | 0,0017615 |
| GO:0045935~positive regulation of nucleobase, nucleoside, nucleotide and nucleic acid metabolic process | 39 | PPARA, CDX2, THRB, ELF4, PAX6, NAA15, NFKB1, ZEB1, RORA, CALR, SRF, HOXD10, IGF1R, BRPF1, MEIS2, NPM2, POU2F1, POU3F2, SHC1, CCNA2, ZNF423, TBL1XR1, IRAK1, KLF12, TGFBR1, MTA2, SMAD4, TP53, SIX4, NR4A3, PPARGC1A, BRCA1, ATXN7L3, NOTCH1, EBF2, FOXC1, NFIC, KLF4, MED1 | 0,0018376 |
| GO:0045449~regulation of transcription | 112 | CDX2, THRB, TLR2, NAA15, BBX, TLR4, RORA, ZNF253, HOXD10, ZFP91, BRPF1, CDKN2A, PHTF2, CCNA2, CCAR1, RREB1, MTA2, ZNF649, SIX4, PPARGC1A, ELL2, PA2G4, NME2, VGLL3, TGFBRAP1, PRDM2, TFAP2C, ERC1, SUV420H1, SUPT6H, MYBL1, MYBL2, CALR, SRF, MEIS1, MEIS2, DMD, HNRNPD, HOXA10, CREBL2, ZNF423, KLF12, TGFBR1, RFX7, KLF11, SMAD4, BRCA2, ZNF521, NR4A3, FOXP4, BRCA1, FOXP2, NOTCH2, NOTCH1, DMTF1, EBF2, JAZF1, TCF12, KLF4, NCOR2, PPARA, BACH2, ELF4, ZFP42, NR6A1, PAX6, DEK, NFKB1, ZEB1, CBFA2T3, BZW1, PCGF3, RHOA, ZNF540, IRAK2, TBL1XR1, IRAK1, KHDRBS3, ZNF90, SOX12, TP53, NCOA7, UBE2I, FLNA, FOXR2, HIPK3, FOXC1, MED1, BTAF1, BCLAF1, SCML2, PML, ZNF367, NR3C1, ZNF654, ZNF660, HIC2, RB1CC1, POU2F1, POU3F2, CHD3, SSRP1, NACC1, SNAI2, SIRT1, ATXN7L3, BCORL1, SP4, ID4, ZBTB2, NFIC, DNM2 | 0,0021625 |
| GO:0010628~positive regulation of gene expression | 36 | PPARA, CDX2, THRB, ELF4, PAX6, NAA15, NFKB1, KIT, ZEB1, RORA, SRF, HOXD10, BRPF1, MEIS2, POU2F1, POU3F2, CCNA2, ZNF423, TBL1XR1, IRAK1, KLF12, TGFBR1, MTA2, SMAD4, TP53, NR4A3, SIX4, PPARGC1A, BRCA1, ATXN7L3, NOTCH1, EBF2, FOXC1, NFIC, KLF4, MED1 | 0,0034838 |
| GO:0006350~transcription | 93 | SCAF1, PPARA, BACH2, CDX2, THRB, ELF4, NR6A1, ZFP42, NAA15, PAX6, BBX, NFKB1, ZEB1, RORA, ZNF253, CBFA2T3, HOXD10, BZW1, ZFP91, BRPF1, PCGF3, CDKN2A, PHTF2, ZNF540, CCAR1, TBL1XR1, KHDRBS3, ZNF90, RREB1, SOX12, TP53, ZNF649, NCOA7, PPARGC1A, ELL2, FOXR2, PA2G4, NME2, HIPK3, VGLL3, FOXC1, PRDM2, TFAP2C, MED1, SUV420H1, SUPT6H, BCLAF1, SCML2, PML, ZNF367, NR3C1, MYBL1, ZNF654, MYBL2, SRF, MEIS1, ZNF660, HIC2, TROVE2, RB1CC1, HOXA10, POU2F1, HNRNPD, POU3F2, GTF3C2, ZNF423, CHD3, CREBL2, SSRP1, NACC1, KLF12, KLF11, SMAD4, ZNF521, NR4A3, SNAI2, FOXP4, SIRT1, ATXN7L3, FOXP2, NOTCH2, PAPOLA, NOTCH1, BCORL1, DMTF1, EBF2, SP4, JAZF1, ZBTB2, NFIC, TCF12, NCOR2, KLF4 | 0,0039425 |
| GO:0045941~positive regulation of transcription | 35 | PPARA, CDX2, THRB, ELF4, PAX6, NAA15, NFKB1, ZEB1, RORA, SRF, HOXD10, BRPF1, MEIS2, POU2F1, POU3F2, CCNA2, ZNF423, TBL1XR1, IRAK1, KLF12, TGFBR1, MTA2, SMAD4, TP53, NR4A3, SIX4, PPARGC1A, BRCA1, ATXN7L3, NOTCH1, EBF2, FOXC1, NFIC, KLF4, MED1 | 0,0040929 |
| GO:0003700~transcription factor activity | 50 | PPARA, CDX2, BACH2, THRB, ELF4, NR6A1, ZFP42, PAX6, NFKB1, RORA, ZEB1, CBFA2T3, HOXD10, CDKN2A, ZNF90, MTA2, TP53, SIX4, FOXR2, NME2, PA2G4, PRDM2, FOXC1, TFAP2C, SUPT6H, BTAF1, SCML2, ZNF367, NR3C1, MYBL2, SRF, MEIS1, MEIS2, POU2F1, HOXA10, POU3F2, CREBL2, ZNF423, KLF12, KLF11, SMAD4, NR4A3, FOXP4, FOXP2, NOTCH1, DMTF1, SP4, NFIC, TCF12, KLF4 | 0,0411494 |
| GO:0016563~transcription activator activity | 27 | PPARA, ELF4, PML, ZEB1, MYBL1, BRPF1, BCL2, HNRNPD, POU2F1, ZNF423, TBL1XR1, IRAK1, RREB1, MTA2, SMAD4, BRCA2, NR4A3, PPARGC1A, BRCA1, ATXN7L3, NOTCH1, SP4, FOXC1, TCF12, KLF4, DNM2, MED1 | 0,0353155 |
| GO:0045944~positive regulation of transcription from RNA polymerase II promoter | 25 | PPARA, THRB, ELF4, PAX6, NFKB1, ZEB1, RORA, SRF, HOXD10, MEIS2, POU2F1, POU3F2, TBL1XR1, KLF12, MTA2, SMAD4, TP53, NR4A3, SIX4, PPARGC1A, NOTCH1, FOXC1, NFIC, KLF4, MED1 | 0,0114797 |
| GO:0045893~positive regulation of transcription, DNA-dependent | 29 | PPARA, CDX2, THRB, ELF4, PAX6, NAA15, NFKB1, ZEB1, RORA, SRF, HOXD10, MEIS2, POU2F1, POU3F2, TBL1XR1, KLF12, MTA2, SMAD4, TP53, NR4A3, SIX4, PPARGC1A, ATXN7L3, BRCA1, NOTCH1, FOXC1, NFIC, KLF4, MED1 | 0,0171215 |
| GO:0051254~positive regulation of RNA metabolic process | 29 | PPARA, CDX2, THRB, ELF4, PAX6, NAA15, NFKB1, ZEB1, RORA, SRF, HOXD10, MEIS2, POU2F1, POU3F2, TBL1XR1, KLF12, MTA2, SMAD4, TP53, NR4A3, SIX4, PPARGC1A, ATXN7L3, BRCA1, NOTCH1, FOXC1, NFIC, KLF4, MED1 | 0,0192325 |
| GO:0003677~DNA binding | 97 | CDX2, THRB, PRR12, BBX, RBM6, RORA, ZNF253, HOXD10, ZFP91, BRPF1, CDKN2A, PHTF2, EGFR, RREB1, MTA2, ZNF649, SIX4, PPARGC1A, TOX3, HNRNPU, PA2G4, NME2, PRDM2, TFAP2C, XRN1, SUPT6H, MYBL1, MYBL2, CALR, SRF, MEIS1, MEIS2, HOXA10, HNRNPD, STRBP, CREBL2, ZNF423, KLF12, SMG6, KLF11, RFX7, SMAD4, BRCA2, ZNF521, NR4A3, FOXP4, BRCA1, FOXP2, DNASE2, NOTCH1, DMTF1, EBF2, TCF12, NCOR2, KLF4, PPARA, KIF22, BACH2, ELF4, ZFP42, NR6A1, PAX6, DEK, NFKB1, ZEB1, CBFA2T3, ZNF540, TBL1XR1, ZNF90, SOX12, TP53, ZFR, FOXR2, HIST2H2BE, FOXC1, MED1, BTAF1, BCLAF1, SCML2, PML, ZNF367, NR3C1, ZNF654, ZNF660, HIC2, SOS1, POU2F1, POU3F2, GTF3C2, CHD3, SSRP1, SNAI2, SNAI1, SON, SP4, ZBTB2, NFIC | 0,0661696 |
| GO:0043565~sequence-specific DNA binding | 34 | PPARA, CDX2, BACH2, THRB, ELF4, NR6A1, PAX6, NFKB1, ZEB1, NR3C1, RORA, SRF, MEIS1, HOXD10, MEIS2, HNRNPD, POU2F1, HOXA10, POU3F2, CREBL2, TBL1XR1, SMG6, MTA2, SMAD4, TP53, SIX4, NR4A3, FOXP4, FOXR2, FOXP2, NOTCH1, FOXC1, TCF12, KLF4 | 0,0702640 |
| GO:0006355~regulation of transcription, DNA-dependent | 74 | PPARA, BACH2, CDX2, THRB, ELF4, NR6A1, NAA15, PAX6, NFKB1, DEK, RORA, ZEB1, ZNF253, CBFA2T3, HOXD10, CDKN2A, RHOA, ZNF540, TBL1XR1, ZNF90, RREB1, MTA2, SOX12, TP53, ZNF649, UBE2I, SIX4, PPARGC1A, FOXR2, PA2G4, NME2, FOXC1, PRDM2, TFAP2C, ERC1, MED1, SUPT6H, ZNF367, MYBL1, NR3C1, CALR, SRF, MEIS1, HIC2, MEIS2, HOXA10, POU2F1, HNRNPD, POU3F2, CHD3, CREBL2, KLF12, RFX7, KLF11, SMAD4, BRCA2, NR4A3, SNAI2, FOXP4, SIRT1, ATXN7L3, BRCA1, FOXP2, NOTCH2, NOTCH1, DMTF1, EBF2, SP4, JAZF1, ID4, NFIC, TCF12, NCOR2, KLF4 | 0,0547518 |
| GO:0051252~regulation of RNA metabolic process | 75 | PPARA, BACH2, CDX2, THRB, ELF4, NR6A1, NAA15, PAX6, NFKB1, DEK, ZEB1, RORA, ZNF253, CBFA2T3, HOXD10, CDKN2A, RHOA, ZNF540, TBL1XR1, ZNF90, RREB1, MTA2, SOX12, TP53, ZNF649, UBE2I, SIX4, PPARGC1A, HNRNPU, FOXR2, PA2G4, NME2, FOXC1, PRDM2, TFAP2C, ERC1, MED1, SUPT6H, ZNF367, MYBL1, NR3C1, CALR, SRF, MEIS1, HIC2, MEIS2, HOXA10, POU2F1, HNRNPD, POU3F2, CHD3, CREBL2, KLF12, RFX7, KLF11, SMAD4, BRCA2, NR4A3, SNAI2, FOXP4, SIRT1, ATXN7L3, BRCA1, FOXP2, NOTCH2, NOTCH1, DMTF1, EBF2, SP4, JAZF1, ID4, NFIC, TCF12, NCOR2, KLF4 | 0,0618061 |
| Cluster 4 - Enrichment Score: 4,119877 |  |  |  |
| GO:0008361~regulation of cell size | 22 | FGFR3, TGFBR1, TP53, SMAD4, PML, MUL1, SOCS5, GREM1, CCNG1, NTN1, ATP2B2, NOTCH2, ZFP91, CDKN1A, CDKN2A, NDRG3, CXCL16, CDKN2AIP, RB1CC1, BCL2, DCUN1D3, SLC9A1 | 0,0003145 |
| GO:0032535~regulation of cellular component size | 24 | FGFR3, TGFBR1, TP53, SMAD4, PML, MUL1, SOCS5, GREM1, CCNG1, NTN1, ARPC1A, ATP2B2, NOTCH2, ZFP91, CDKN1A, CDKN2A, NDRG3, CXCL16, CDKN2AIP, RB1CC1, BCL2, RAC1, DCUN1D3, SLC9A1 | 0,0011282 |
| GO:0030308~negative regulation of cell growth | 12 | CDKN1A, CDKN2A, NDRG3, BCL2, CDKN2AIP, PML, SMAD4, TP53, MUL1, DCUN1D3, GREM1, NTN1 | 0,0041050 |
| GO:0045792~negative regulation of cell size | 12 | CDKN1A, CDKN2A, NDRG3, BCL2, CDKN2AIP, PML, SMAD4, TP53, MUL1, DCUN1D3, GREM1, NTN1 | 0,0069562 |
| GO:0040008~regulation of growth | 24 | TGFBR1, TGFBR2, TP53, SMAD4, PML, MUL1, SOCS5, GREM1, CXADR, NTN1, BCL2L11, NRCAM, ZFP91, UBE2E3, CDKN1A, CDKN2A, NDRG3, CXCL16, CDKN2AIP, BCL2, POU3F2, FOXC1, SHC1, DCUN1D3 | 0,0091658 |
| GO:0045926~negative regulation of growth | 12 | CDKN1A, CDKN2A, NDRG3, BCL2, CDKN2AIP, PML, SMAD4, TP53, MUL1, DCUN1D3, GREM1, NTN1 | 0,0144128 |
| GO:0001558~regulation of cell growth | 16 | TGFBR1, TP53, SMAD4, PML, MUL1, GREM1, NTN1, NRCAM, ZFP91, CDKN1A, CDKN2A, NDRG3, CXCL16, CDKN2AIP, BCL2, DCUN1D3 | 0,0211964 |
| Cluster 5 - Enrichment Score: 3,996915 |  |  |  |
| GO:0048667~cell morphogenesis involved in neuron differentiation | 20 | NRP2, WNT3A, PAX6, CELSR3, NTN1, SLIT3, NRCAM, ALCAM, ATP2B2, IGF1R, SLITRK4, NOTCH1, BDNF, CXCR4, CLIC5, BCL2, RAC1, NUMB, NGFR, SNAP25 | 0,0015093 |
| GO:0048812~neuron projection morphogenesis | 20 | EGFR, NRP2, WNT3A, PAX6, CELSR3, NTN1, SLIT3, NRCAM, ALCAM, IGF1R, SLITRK4, NOTCH1, BDNF, CXCR4, DMD, BCL2, RAC1, NUMB, NGFR, SNAP25 | 0,0017975 |
| GO:0030182~neuron differentiation | 31 | NRP2, SEPT2, WNT3A, PAX6, RORA, SRF, HOXD10, NRCAM, ALCAM, ATP2B2, ZFP91, IGF1R, BDNF, CXCR4, DMD, BCL2, RAC1, NUMB, POU3F2, SNAP25, EGFR, TGFBR1, SMAD4, CELSR3, NTN1, SLIT3, NOTCH1, SLITRK4, CLIC5, ID4, NGFR | 0,0017651 |
| GO:0032990~cell part morphogenesis | 22 | EGFR, NRP2, COX10, WNT3A, PAX6, CELSR3, MUL1, NTN1, SLIT3, NRCAM, ALCAM, IGF1R, SLITRK4, NOTCH1, BDNF, CXCR4, DMD, BCL2, RAC1, NUMB, NGFR, SNAP25 | 0,0019510 |
| GO:0048666~neuron development | 26 | NRP2, SEPT2, WNT3A, PAX6, SRF, HOXD10, NRCAM, ALCAM, IGF1R, ZFP91, ATP2B2, BDNF, CXCR4, DMD, BCL2, RAC1, NUMB, SNAP25, EGFR, CELSR3, NTN1, SLIT3, NOTCH1, SLITRK4, CLIC5, NGFR | 0,0020470 |
| GO:0000904~cell morphogenesis involved in differentiation | 21 | NRP2, WNT3A, PAX6, CELSR3, NTN1, SLIT3, NRCAM, ALCAM, ATP2B2, IGF1R, SLITRK4, NOTCH1, BDNF, CXCR4, CLIC5, BCL2, RAC1, NUMB, NGFR, LAMC1, SNAP25 | 0,0026631 |
| GO:0007409~axonogenesis | 18 | NRP2, WNT3A, PAX6, CELSR3, NTN1, SLIT3, NRCAM, ALCAM, IGF1R, SLITRK4, NOTCH1, BDNF, CXCR4, BCL2, RAC1, NUMB, NGFR, SNAP25 | 0,0034542 |
| GO:0031175~neuron projection development | 21 | EGFR, NRP2, SEPT2, WNT3A, PAX6, CELSR3, NTN1, SLIT3, NRCAM, ALCAM, IGF1R, SLITRK4, NOTCH1, BDNF, CXCR4, DMD, BCL2, RAC1, NUMB, NGFR, SNAP25 | 0,0041698 |
| GO:0048858~cell projection morphogenesis | 20 | EGFR, NRP2, WNT3A, PAX6, CELSR3, NTN1, SLIT3, NRCAM, ALCAM, IGF1R, SLITRK4, NOTCH1, BDNF, CXCR4, DMD, BCL2, RAC1, NUMB, NGFR, SNAP25 | 0,0061322 |
| GO:0032989~cellular component morphogenesis | 27 | NRP2, COX10, WNT3A, PAX6, NRCAM, ALCAM, ATP2B2, IGF1R, BDNF, EZR, CXCR4, DMD, BCL2, RAC1, NUMB, RHOA, SNAP25, EGFR, CELSR3, MUL1, NTN1, SLIT3, NOTCH1, SLITRK4, CLIC5, NGFR, LAMC1 | 0,0064783 |
| GO:0000902~cell morphogenesis | 25 | EGFR, NRP2, WNT3A, PAX6, CELSR3, NTN1, SLIT3, NRCAM, ALCAM, ATP2B2, IGF1R, SLITRK4, NOTCH1, BDNF, EZR, CXCR4, CLIC5, DMD, BCL2, RAC1, NUMB, RHOA, NGFR, LAMC1, SNAP25 | 0,0071883 |
| GO:0030030~cell projection organization | 25 | EGFR, NRP2, CCDC88A, SEPT2, ROCK1, WNT3A, PAX6, CELSR3, NTN1, SLIT3, NRCAM, ALCAM, ATP2B2, IGF1R, SLITRK4, NOTCH1, BDNF, CXCR4, CLIC5, DMD, BCL2, RAC1, NUMB, NGFR, SNAP25 | 0,0107583 |
| GO:0007411~axon guidance | 9 | NRP2, ALCAM, BDNF, CXCR4, RAC1, PAX6, NGFR, NTN1, SLIT3 | 0,1847677 |
| Cluster 6 - Enrichment Score: 3,723315 |  |  |  |
| GO:0005626~insoluble fraction | 45 | NRP2, SEPT2, GCNT2, CADM1, SLC20A2, ADCY6, PTGS1, PML, FKBP1A, CALR, SYP, ATP2B2, IGF1R, BCL2, DMD, CYP7A1, RAC1, ELOVL2, SNPH, ACSL4, PLCB1, TRAF6, SLC4A3, NT5E, FRS2, SNAP25, SLC12A6, RAB8B, MAN1A2, CLCC1, TP53, CELSR3, GRIA3, APPL1, BCL2L11, ITPR1, SLIT3, NME2, H6PD, SLC7A2, IGF2R, CLIC5, CNTN1, VAMP2, SLC9A1 | 0,0039856 |
| GO:0000267~cell fraction | 51 | NRP2, SEPT2, CADM1, SLC20A2, ADCY6, PTGS1, SYP, ATP2B2, GSR, CYP7A1, ELOVL2, SNPH, FAS, PLCB1, NT5E, FRS2, SLC4A3, SLC12A6, MAN1A2, TP53, BCL2L11, SLIT3, NME2, CLIC4, CD40LG, H6PD, TPPP, CLIC5, IGF2R, CNTN1, VAMP2, YWHAZ, GCNT2, PML, FKBP1A, CALR, IGF1R, BCL2, DMD, RAC1, ACSL4, TRAF6, SNAP25, RAB8B, CLCC1, CELSR3, GRIA3, APPL1, ITPR1, SLC7A2, SLC9A1 | 0,0127010 |
| GO:0005624~membrane fraction | 41 | NRP2, GCNT2, SEPT2, CADM1, SLC20A2, ADCY6, PTGS1, FKBP1A, CALR, SYP, ATP2B2, IGF1R, CYP7A1, DMD, BCL2, RAC1, ELOVL2, SNPH, ACSL4, PLCB1, TRAF6, SLC4A3, NT5E, FRS2, SNAP25, SLC12A6, MAN1A2, CLCC1, CELSR3, GRIA3, APPL1, ITPR1, BCL2L11, SLIT3, NME2, H6PD, SLC7A2, IGF2R, CNTN1, VAMP2, SLC9A1 | 0,0116162 |
| Cluster 7 - Enrichment Score: 3,597052 |  |  |  |
| GO:0045596~negative regulation of cell differentiation | 24 | PPARA, CDX2, TGFBR1, NF1, TP53, PAX6, TLR4, KIT, SOCS5, ZEB1, CALR, NTN1, SIRT1, NOTCH2, ZFP91, NME2, NOTCH1, BDNF, RHOA, GDF11, ID4, NGFR, KLF4, RC3H1 | 0,0000979 |
| GO:0051960~regulation of nervous system development | 21 | NF1, NLGN1, TP53, PAX6, KIT, CDH2, CALR, NTN1, NRCAM, ZFP91, NOTCH1, BDNF, TIAM1, BCL2, NUMB, RHOA, POU3F2, ID4, NGFR, SNAP25, EIF2AK3 | 0,0003629 |
| GO:0050767~regulation of neurogenesis | 19 | NF1, NLGN1, TP53, PAX6, KIT, CDH2, CALR, NTN1, NRCAM, ZFP91, NOTCH1, BDNF, TIAM1, BCL2, NUMB, RHOA, POU3F2, ID4, NGFR | 0,0006270 |
| GO:0045664~regulation of neuron differentiation | 16 | NLGN1, PAX6, CDH2, CALR, NTN1, NRCAM, ZFP91, NOTCH1, BDNF, TIAM1, BCL2, NUMB, RHOA, POU3F2, ID4, NGFR | 0,0012172 |
| GO:0060284~regulation of cell development | 20 | NF1, NLGN1, TP53, SMAD4, PAX6, KIT, CDH2, CALR, NTN1, NRCAM, ZFP91, NOTCH1, BDNF, TIAM1, BCL2, NUMB, RHOA, POU3F2, ID4, NGFR | 0,0012666 |
| GO:0045597~positive regulation of cell differentiation | 21 | CDX2, THRB, TGFBR2, SMAD4, PAX6, NFKB1, KIT, SOCS5, SRF, NTN1, NRCAM, ZFP91, NME2, NOTCH1, BDNF, TIAM1, BCL2, NUMB, RHOA, CAMK1, NGFR | 0,0015935 |
| GO:0051130~positive regulation of cellular component organization | 18 | TGFBR1, SMAD4, PML, APOC2, KIT, CALR, SRF, PPARGC1A, NTN1, ZFP91, TIAM1, TPPP, NUMB, RAC1, RHOA, FAF1, NGFR, EIF5A2 | 0,0019885 |
| GO:0010720~positive regulation of cell development | 11 | ZFP91, NOTCH1, TIAM1, BCL2, NUMB, RHOA, PAX6, SMAD4, KIT, NGFR, NTN1 | 0,0021817 |
| GO:0031346~positive regulation of cell projection organization | 9 | ZFP91, TIAM1, TGFBR1, NUMB, RAC1, RHOA, KIT, NGFR, NTN1 | 0,0035164 |
| GO:0031344~regulation of cell projection organization | 12 | NRCAM, ZFP91, TIAM1, TGFBR1, NUMB, RAC1, RHOA, POU3F2, KIT, NGFR, CDH2, NTN1 | 0,0034091 |
| GO:0051094~positive regulation of developmental process | 21 | CDX2, THRB, TGFBR2, SMAD4, PAX6, NFKB1, KIT, SOCS5, SRF, NTN1, NRCAM, ZFP91, NME2, NOTCH1, BDNF, TIAM1, BCL2, NUMB, RHOA, CAMK1, NGFR | 0,0096938 |
| GO:0050769~positive regulation of neurogenesis | 9 | ZFP91, NOTCH1, TIAM1, NUMB, RHOA, PAX6, KIT, NGFR, NTN1 | 0,0115157 |
| GO:0010769~regulation of cell morphogenesis involved in differentiation | 10 | NRCAM, ZFP91, TIAM1, NUMB, RHOA, SMAD4, POU3F2, NGFR, CDH2, NTN1 | 0,0147801 |
| GO:0010975~regulation of neuron projection development | 9 | NRCAM, ZFP91, TIAM1, NUMB, RHOA, POU3F2, NGFR, CDH2, NTN1 | 0,0293886 |
| GO:0050770~regulation of axonogenesis | 8 | NRCAM, ZFP91, TIAM1, RHOA, POU3F2, NGFR, CDH2, NTN1 | 0,0362622 |
| GO:0022604~regulation of cell morphogenesis | 12 | NRCAM, ALDOA, ZFP91, EZR, TIAM1, NUMB, RHOA, SMAD4, POU3F2, NGFR, CDH2, NTN1 | 0,0422311 |
| GO:0045665~negative regulation of neuron differentiation | 6 | ZFP91, NOTCH1, RHOA, PAX6, ID4, CALR | 0,0533476 |
| GO:0050772~positive regulation of axonogenesis | 5 | ZFP91, TIAM1, RHOA, NGFR, NTN1 | 0,0938521 |
| GO:0050771~negative regulation of axonogenesis | 3 | RHOA, NGFR, NTN1 | 0,6986504 |
| GO:0031345~negative regulation of cell projection organization | 3 | RHOA, NGFR, NTN1 | 0,7532896 |
| GO:0051129~negative regulation of cellular component organization | 7 | RAC1, RHOA, APOC2, NGFR, MAPRE1, NTN1, BRCA1 | 0,8124174 |
| Cluster 8 - Enrichment Score: 3,1698553 |  |  |  |
| GO:0005654~nucleoplasm | 47 | CDX2, MCL1, ELF4, NR6A1, PML, NAA15, INTS2, NFKB1, ZEB1, CBFA2T3, SF3B1, BRPF1, CDKN2A, NUP50, HOXA10, POU2F1, POU3F2, GTF3C2, CCNA2, NMD3, CHD3, SSRP1, CDC6, TBL1XR1, NACC1, RREB1, MTA2, SMAD4, TP53, BRCA2, UBE2I, APPL1, SIRT1, PPARGC1A, BRCA1, ATXN7L3, ELL2, SON, CDKN1A, CDKN2AIP, JAZF1, CALM3, NGFR, TCF12, KLF4, NCOR2, MED1 | 0,0050505 |
| GO:0031981~nuclear lumen | 67 | KDM6A, CDX2, SEPT2, ELF4, NR6A1, NAA15, INTS2, NFKB1, ZEB1, CBFA2T3, BRPF1, CDKN2A, ZNF540, CCNA2, TBL1XR1, CDC6, KHDRBS3, RREB1, MTA2, TP53, UBE2I, ZFR, RBBP6, PPARGC1A, ELL2, PA2G4, NGFR, MED1, MCL1, TXN2, PML, MYBL1, NR3C1, KIT, SRF, DAZAP1, SF3B1, MEIS2, NUP50, HOXA10, POU2F1, POU3F2, GTF3C2, NOVA1, NMD3, CHD3, SSRP1, NACC1, SMG6, SMAD4, BRCA2, APPL1, FOXP4, SIRT1, ITPR1, ATXN7L3, BRCA1, PAPOLA, SON, CDKN1A, CDKN2AIP, JAZF1, CALM3, SH3D19, TCF12, NCOR2, KLF4 | 0,0035802 |
| GO:0044451~nucleoplasm part | 32 | CDX2, ELF4, NR6A1, PML, NAA15, INTS2, ZEB1, SF3B1, BRPF1, POU2F1, HOXA10, POU3F2, GTF3C2, CHD3, TBL1XR1, NACC1, RREB1, MTA2, SMAD4, TP53, UBE2I, APPL1, SIRT1, PPARGC1A, ATXN7L3, ELL2, SON, JAZF1, TCF12, KLF4, NCOR2, MED1 | 0,0101943 |
| GO:0070013~intracellular organelle lumen | 72 | KDM6A, CDX2, SEPT2, ELF4, NR6A1, NAA15, INTS2, NFKB1, ZEB1, CBFA2T3, BRPF1, CDKN2A, P4HA1, ZNF540, CCNA2, TBL1XR1, CDC6, KHDRBS3, RREB1, MTA2, TP53, UBE2I, ZFR, PPARGC1A, RBBP6, ELL2, PA2G4, H6PD, PPM1K, NGFR, MED1, MCL1, TXN2, PML, MYBL1, NR3C1, KIT, CALR, SRF, DAZAP1, SF3B1, MEIS2, NUP50, HOXA10, POU2F1, POU3F2, GTF3C2, NOVA1, CHD3, NMD3, SSRP1, NACC1, SMG6, SMAD4, GOLIM4, BRCA2, APPL1, FOXP4, SIRT1, ITPR1, ATXN7L3, BRCA1, PAPOLA, SON, CDKN1A, CDKN2AIP, JAZF1, CALM3, SH3D19, TCF12, NCOR2, KLF4 | 0,0261367 |
| GO:0043233~organelle lumen | 72 | KDM6A, CDX2, SEPT2, ELF4, NR6A1, NAA15, INTS2, NFKB1, ZEB1, CBFA2T3, BRPF1, CDKN2A, P4HA1, ZNF540, CCNA2, TBL1XR1, CDC6, KHDRBS3, RREB1, MTA2, TP53, UBE2I, ZFR, PPARGC1A, RBBP6, ELL2, PA2G4, H6PD, PPM1K, NGFR, MED1, MCL1, TXN2, PML, MYBL1, NR3C1, KIT, CALR, SRF, DAZAP1, SF3B1, MEIS2, NUP50, HOXA10, POU2F1, POU3F2, GTF3C2, NOVA1, CHD3, NMD3, SSRP1, NACC1, SMG6, SMAD4, GOLIM4, BRCA2, APPL1, FOXP4, SIRT1, ITPR1, ATXN7L3, BRCA1, PAPOLA, SON, CDKN1A, CDKN2AIP, JAZF1, CALM3, SH3D19, TCF12, NCOR2, KLF4 | 0,0374980 |
| GO:0031974~membrane-enclosed lumen | 73 | KDM6A, CDX2, SEPT2, ELF4, NR6A1, NAA15, INTS2, NFKB1, ZEB1, CBFA2T3, BRPF1, CDKN2A, P4HA1, ZNF540, CCNA2, TBL1XR1, CDC6, KHDRBS3, RREB1, MTA2, TP53, UBE2I, ZFR, PPARGC1A, RBBP6, ELL2, PA2G4, H6PD, PPM1K, IGF2R, NGFR, MED1, MCL1, TXN2, PML, MYBL1, NR3C1, KIT, CALR, SRF, DAZAP1, SF3B1, MEIS2, NUP50, HOXA10, POU2F1, POU3F2, GTF3C2, NOVA1, CHD3, NMD3, SSRP1, NACC1, SMG6, SMAD4, GOLIM4, BRCA2, APPL1, FOXP4, SIRT1, ITPR1, ATXN7L3, BRCA1, PAPOLA, SON, CDKN1A, CDKN2AIP, JAZF1, CALM3, SH3D19, TCF12, NCOR2, KLF4 | 0,0373429 |
| GO:0005730~nucleolus | 31 | KDM6A, SEPT2, TXN2, PML, KIT, NR3C1, MYBL1, CBFA2T3, SRF, DAZAP1, MEIS2, CDKN2A, ZNF540, NOVA1, NMD3, NACC1, KHDRBS3, SMG6, MTA2, TP53, SIRT1, ZFR, FOXP4, RBBP6, ITPR1, PAPOLA, PA2G4, CDKN2AIP, SH3D19, NCOR2, KLF4 | 0,1267692 |
| Cluster 9 - Enrichment Score: 3,043012 |  |  |  |
| GO:0016192~vesicle-mediated transport | 37 | PACS1, YWHAZ, RIMS4, HOOK3, SYP, AP1S3, AP1S1, AP1S2, AP3M1, ACTR1A, RAC1, SNPH, SEC22C, SNAP25, SEC24D, RHOBTB3, FAM160A2, TGFBR2, NLGN1, MON2, FLNA, CORO1C, NME2, AP2A2, GAPVD1, FNBP1L, STXBP5, IGF2R, CXCL16, ARCN1, RAB22A, YIPF5, SH3D19, VAMP2, ERC1, GGA2, DNM2 | 0,0018360 |
| GO:0016044~membrane organization | 25 | NRCAM, AP1S3, SYP, AP1S1, AP1S2, BCL2, RAC1, SEC24D, EGFR, CCDC88A, TGFBR2, TP53, REEP1, CORO1C, AP2A2, NME2, GAPVD1, FNBP1L, CXCL16, IGF2R, ARCN1, RAB22A, SH3D19, VAMP2, DNM2 | 0,0160333 |
| GO:0006897~endocytosis | 15 | TGFBR2, AP1S3, CORO1C, SYP, AP2A2, NME2, AP1S1, AP1S2, GAPVD1, FNBP1L, CXCL16, IGF2R, RAB22A, RAC1, DNM2 | 0,1006163 |
| GO:0010324~membrane invagination | 15 | TGFBR2, AP1S3, CORO1C, SYP, AP2A2, NME2, AP1S1, AP1S2, GAPVD1, FNBP1L, CXCL16, IGF2R, RAB22A, RAC1, DNM2 | 0,1006163 |
| Cluster 10 - Enrichment Score: 3,0075646 |  |  |  |
| hsa05200:Pathways in cancer | 26 | FGFR3, WNT3A, PML, NFKB1, KIT, IGF1R, CDKN2A, BCL2, SOS1, RAC1, RHOA, FAS, TRAF6, EGFR, IL8, TGFBR1, TGFBR2, SMAD4, TP53, BRCA2, FADD, APPL1, BIRC2, CCDC6, CDKN1A, LAMC1 | 0,0084127 |
| hsa05210:Colorectal cancer | 10 | EGFR, IGF1R, BCL2, SOS1, TGFBR1, TGFBR2, RAC1, SMAD4, TP53, APPL1 | 0,0937411 |
| hsa05212:Pancreatic cancer | 9 | EGFR, CDKN2A, TGFBR1, TGFBR2, RAC1, SMAD4, TP53, BRCA2, NFKB1 | 0,0973067 |
| Cluster 10 - Enrichment Score: 2,9346208 |  |  |  |
| GO:0051174~regulation of phosphorus metabolic process | 33 | CDK5R2, ADCY6, TLR4, FKBP1A, KIT, CCNG1, ZFP91, DUSP19, CDKN2A, CXCR4, BCL2, RB1CC1, RAC1, SHC1, SPRED1, TRAF6, FRS2, EGFR, CDC6, IRAK1, CCDC88A, SMG6, TGFBR1, NF1, TGFBR2, SMAD4, MUL1, CDKN3, GRM4, SPAG9, CDKN1A, HIPK3, DUSP8 | 0,0017924 |
| GO:0019220~regulation of phosphate metabolic process | 33 | CDK5R2, ADCY6, TLR4, FKBP1A, KIT, CCNG1, ZFP91, DUSP19, CDKN2A, CXCR4, BCL2, RB1CC1, RAC1, SHC1, SPRED1, TRAF6, FRS2, EGFR, CDC6, IRAK1, CCDC88A, SMG6, TGFBR1, NF1, TGFBR2, SMAD4, MUL1, CDKN3, GRM4, SPAG9, CDKN1A, HIPK3, DUSP8 | 0,0017924 |
| GO:0042325~regulation of phosphorylation | 32 | CDK5R2, ADCY6, FKBP1A, TLR4, KIT, CCNG1, ZFP91, DUSP19, CDKN2A, CXCR4, BCL2, RB1CC1, RAC1, SHC1, SPRED1, TRAF6, FRS2, EGFR, CDC6, IRAK1, CCDC88A, TGFBR1, NF1, TGFBR2, SMAD4, MUL1, CDKN3, GRM4, SPAG9, CDKN1A, HIPK3, DUSP8 | 0,0019275 |
| GO:0051338~regulation of transferase activity | 27 | CDK5R2, ADCY6, KIT, CCNG1, SERINC5, DUSP19, CDKN2A, CXCR4, RAC1, SHC1, SPRED1, TRAF6, FRS2, EGFR, CDC6, IRAK1, CCDC88A, TGFBR1, NF1, TGFBR2, MUL1, CDKN3, GRM4, SPAG9, CDKN1A, HIPK3, DUSP8 | 0,0031767 |
| GO:0043549~regulation of kinase activity | 26 | CDK5R2, ADCY6, KIT, CCNG1, DUSP19, CDKN2A, CXCR4, RAC1, SHC1, SPRED1, TRAF6, FRS2, EGFR, CDC6, IRAK1, CCDC88A, TGFBR1, NF1, TGFBR2, MUL1, CDKN3, GRM4, SPAG9, CDKN1A, HIPK3, DUSP8 | 0,0036793 |
| GO:0045859~regulation of protein kinase activity | 25 | CDK5R2, ADCY6, KIT, CCNG1, DUSP19, CDKN2A, CXCR4, SHC1, SPRED1, TRAF6, FRS2, EGFR, CDC6, IRAK1, CCDC88A, TGFBR1, TGFBR2, NF1, MUL1, CDKN3, GRM4, SPAG9, CDKN1A, HIPK3, DUSP8 | 0,0051128 |
| GO:0007243~protein kinase cascade | 26 | FGFR3, TLR2, MKNK2, TLR4, KIT, IGF1R, TMED7, DUSP19, CXCR4, RB1CC1, SHC1, SPRED1, TRAF6, FRS2, IRAK2, EGFR, IRAK1, TGFBR1, NF1, MUL1, SPAG9, GRM4, FAF1, ERC1, DUSP8, SLC9A1 | 0,0057762 |
| GO:0044093~positive regulation of molecular function | 34 | ADCY6, GNG13, PML, TLR2, APOC2, TLR4, FKBP1A, KIT, SLC11A2, SERINC5, CDKN2A, CXCR4, BCL2, NPM2, RAC1, TGM2, SHC1, TRAF6, FRS2, IRAK2, EGFR, IRAK1, CCDC88A, TGFBR1, NF1, TGFBR2, TP53, MUL1, GRM4, SPAG9, NME2, CALM3, ERC1, EIF2AK3 | 0,0126331 |
| GO:0043405~regulation of MAP kinase activity | 13 | EGFR, NF1, MUL1, KIT, SPAG9, GRM4, DUSP19, CXCR4, HIPK3, SHC1, SPRED1, DUSP8, FRS2 | 0,0289690 |

**Supplementary Table S4.** Target genes for each deferentially expressed miRNA.

| **miRNA** | **Targets** |
| --- | --- |
| hsa-miR-133a-3p | RHOA, CDC42, ERG, HCN4, UCP2, KRT7, CACNA1C, HCN2, PKM, CASP9, KCNQ1, FSCN1, KCNH2, TAGLN2, LASP1, PNP, MSN, EGFR, VKORC1, PRDM16 |
| hsa-miR-122-5p | CYP7A1, IGF1R, SRF, RAC1, RHOA, CCNG1 |
| hsa-miR-126-5p | SLC45A3, PTPN7, ADAM9, MMP7, CXCL12 |
| hsa-miR-10b-5p | HOXD10, KLF4, PPARA, NCOR2, NF1, PIEZO1, BCL2L11, TFAP2C, CDKN2A, CDKN1A, TRA2B, SRSF1, TP53, NOTCH1, PAX6, MAPRE1, SDC1, NR4A3, NRP2 |
| hsa-miR-204-5p | MEIS1, HOXA10, MCL1, BCL2, TGFBR1, TGFBR2, SNAI2, MEIS2, SNAI1, NBR1, THRB, CDX2, AKAP1, AP1S1, AP1S2, ARHGAP29, ATP2B1, BCL2L2, BIRC2, CDH2, COL5A3, CXCL3, EDEM1, EZR |
| hsa-miR-148a-5p | GALK2, ACTR1A, CALR, ID4, SYAP1 |
| hsa-miR-146a-5p | CXCR4, KIT, CFH, IRAK2, TLR2, FADD, TRAF6, IRAK1, ROCK1, BRCA2, BRCA1, FAF1, CCNA2, PA2G4, SNAP25, IL8, NFKB1, CDKN1A, EGFR, MTA2, CD40LG, FAS, CDKN3, KIF22, ERBB4, SMAD4, TLR4 |
| hsa-miR-423-5p | RABAC1, NMD3, YWHAZ, RGPD2, TSPYL6, HIST2H2BE, USP6, PGAM4, DIP2A, NOS1AP, IGDCC4, RC3H1, KIF1A, SRSF11, BCLAF1, RPS15A, CCNI, GDF11, FAM127A, NME2, COX1, ARPC1A, MYBL2, ND5 |
